# Effective connectivity during face processing in major depression – distinguishing markers of pathology, risk, and resilience

**DOI:** 10.1017/S0033291722000824

**Published:** 2023-07

**Authors:** Seda Sacu, Carolin Wackerhagen, Susanne Erk, Nina Romanczuk-Seiferth, Kristina Schwarz, Janina I. Schweiger, Heike Tost, Andreas Meyer-Lindenberg, Andreas Heinz, Adeel Razi, Henrik Walter

**Affiliations:** 1 Berlin School of Mind and Brain, Humboldt-Universität zu Berlin, Berlin, Germany; 2 Department of Child and Adolescent Psychiatry and Psychotherapy, Central Institute of Mental Health, Medical Faculty Mannheim, Heidelberg University, Mannheim, Germany; 3 Division of Mind and Brain Research, Department of Psychiatry and Psychotherapy CCM, Charité – Universitätsmedizin Berlin, corporate member of Freie Universität Berlin, Humboldt-Universität zu Berlin, and Berlin Institute of Health, Berlin, Germany; 4 Systems Neuroscience in Psychiatry, Department of Psychiatry and Psychotherapy, Central Institute of Mental Health, Medical Faculty Mannheim, Heidelberg University, Mannheim, Germany; 5 Wellcome Centre for Human Neuroimaging, Institute of Neurology, University College London, London, UK; 6 Turner Institute for Brain and Mental Health & Monash Biomedical Imaging, Monash University, Clayton, Australia

**Keywords:** Effective connectivity, emotional face processing, familial risk, fMRI, major depressive disorder, resilience

## Abstract

**Background:**

Aberrant brain connectivity during emotional processing, especially within the fronto-limbic pathway, is one of the hallmarks of major depressive disorder (MDD). However, the methodological heterogeneity of previous studies made it difficult to determine the functional and etiological implications of specific alterations in brain connectivity. We previously reported alterations in psychophysiological interaction measures during emotional face processing, distinguishing depressive pathology from at-risk/resilient and healthy states. Here, we extended these findings by effective connectivity analyses in the same sample to establish a refined neural model of emotion processing in depression.

**Methods:**

Thirty-seven patients with MDD, 45 first-degree relatives of patients with MDD and 97 healthy controls performed a face-matching task during functional magnetic resonance imaging. We used dynamic causal modeling to estimate task-dependent effective connectivity at the subject level. Parametric empirical Bayes was performed to quantify group differences in effective connectivity.

**Results:**

MDD patients showed decreased effective connectivity from the left amygdala and left lateral prefrontal cortex to the fusiform gyrus compared to relatives and controls, whereas patients and relatives showed decreased connectivity from the right orbitofrontal cortex to the left insula and from the left orbitofrontal cortex to the right fusiform gyrus compared to controls. Relatives showed increased connectivity from the anterior cingulate cortex to the left dorsolateral prefrontal cortex compared to patients and controls.

**Conclusions:**

Our results suggest that the depressive state alters top-down control of higher visual regions during face processing. Alterations in connectivity within the cognitive control network present potential risk or resilience mechanisms.

## Introduction

Major depressive disorder (MDD) is a common and debilitating mental health problem with a well-known familial association (Sullivan, Neale, & Kendler, 2000). Due to the contribution of both genetic and environmental factors, individuals with a family history of MDD are at a greater risk for developing depression compared to those without a family history of MDD (Klein et al., 2013; Li, Sundquist, Hemminki, & Sundquist, 2008; Weissman et al., 2006; Wilde et al., 2014). However, the pathway from familial risk to clinical symptoms has not been fully understood in MDD.

Through the elevated risk of developing depression, unaffected first-degree relatives are assumed to share some biological and psychological features with MDD patients (Hasler, Drevets, Manji, & Charney, 2004; Meyer-Lindenberg & Weinberger, 2006). Altered fronto-limbic connectivity is one of these putative risk markers which is associated with MDD and present independently from clinical status (Fornito & Bullmore, 2012). Several studies showed that both depressed patients (Carballedo et al., 2011; Chen et al., 2008; Dannlowski et al., 2009; De Almeida et al., 2011; Kong et al., 2013; Lu et al., 2012; Matthews, Strigo, Simmons, Yang, & Paulus, 2008; Moses-Kolko et al., 2010) and individuals with a family history of depression (Miskowiak et al., 2015, 2017; Wackerhagen et al., 2017) exhibited decreased connectivity in the fronto-limbic pathway during the processing of facial emotion. Although these results support that abnormal fronto-limbic connectivity can be a risk marker for MDD, previous studies compared either MDD patients or individuals at familial risk for MDD with healthy controls. Therefore, it is not clear to what extent alterations in the fronto-limbic pathways are shared by patients and individuals at familial risk for MDD. A systematic approach is, thus, necessary to dissociate disease-specific and risk-related alterations in this circuitry.

On the other hand, despite the familial risk for psychiatric disorders, which is comprised of both genetic and environmental factors, many first-degree relatives do not develop depression and stay psychologically healthy. This phenomenon can be described as resilience, i.e. the maintenance of mental health despite adversity (Kalisch et al., 2017; Rodman, Jenness, Weissman, Pine, & McLaughlin, 2019). It is possible that neural or neurodevelopmental mechanisms in healthy relatives enable this resilience capacity, and that these are unique to the relatives group compared to both patients and controls. Given the high prevalence rate and poor treatment outcomes for MDD (Eckshtain et al., 2020; Kessler & Bromet, 2013), identifying biopsychosocial factors promoting resilience, as well as the neural mechanism underlying it, becomes important to develop novel approaches to the prevention and treatment of depression (Holz, Tost, & Meyer-Lindenberg, 2020).

Although research into the neuroscience of resilience is relatively new, previous studies have suggested that brain regions involved in cognitive control of emotions are associated with resilience to depression (Gupta et al., 2017; Rodman et al., 2019). One of these brain regions associated with resilience capacity is the anterior cingulate cortex (Holz et al., 2020). Previous neuroimaging studies showed that resilience to depression is linked to greater anterior cingulate cortex volume (Gupta et al., 2017; Holz et al., 2016), greater anterior cingulate cortex activation during a cognitive task (Peterson et al., 2014) and enhanced connectivity between anterior cingulate cortex and the prefrontal cortex during the processing of emotional faces (Wackerhagen et al., 2019, 2017). Taken together, these findings suggest that the connectivity of the anterior cingulate cortex may be a promising target to test resilience mechanism for MDD.

Here, we re-assessed data reported by Wackerhagen et al. (2019) and adopted the same group-comparative approach (online Supplementary material S1) to disentangle disease, risk, and resilience in neuro-functional markers for MDD during an implicit emotion processing task. However, instead of using generalized psychophysiological interactions (PPIs) (McLaren, Ries, Xu, & Johnson, 2012) to assess task-dependent functional connectivity, we utilized dynamic causal modeling (DCM) to measure effective connectivity in the target neural pathways. This brings two main methodological advantages to the present study. First, unlike functional connectivity, effective connectivity allows us to determine the direction of influences (i.e. causal relationships in the context of the model) as well as the valence of the influence (i.e. inhibitory or excitatory signaling) among coupled brain regions (Friston, 2011; Friston, Harrison, & Penny, 2003). Second, the majority of task-dependent functional connectivity studies used amygdala as a seed region and investigated the amygdala connectivity with the rest of the brain in MDD patients and individuals at high risk for MDD. Although the amygdala plays a crucial role in depression, we wanted to allow for new insights into the neural model of MDD by utilizing a model-based approach that takes into account the connectivity patterns of other important brain regions, such as the prefrontal cortex (Koenigs et al., 2008; Pizzagalli, 2011) and high-level visual regions like the fusiform gyrus (Ho et al., 2016; Li et al., 2013; Townsend et al., 2010).

Based on our previous study (Wackerhagen et al., 2019), we hypothesized that decreased effective connectivity within the fronto-limbic pathway will be identified as disease and/or risk factor for MDD, whereas connectivity between anterior cingulate cortex and prefrontal cortex will be related to resilience to MDD. Additionally, there is a growing literature showing that MDD patients have functional abnormalities in visual cortex regions during emotional face processing (Alders et al., 2019; Colich, Foland-Ross, Eggleston, Singh, & Gotlib, 2016; Furey et al., 2013; Ho et al., 2016; Li et al., 2018, 2013; Townsend et al., 2010). Therefore, we wanted to explore whether altered effective connectivity of fusiform gyrus will be associated with disease state and/or risk for MDD. Finally, we explored the relationship between negative affect and task-related connectivity to evaluate the functional relevance of potential alterations.

## Methods and materials

### Participants

Forty-eight patients with MDD (34 females, mean age = 31.25), 49 first-degree relatives of patients with MDD (33 females, mean age = 28.49), and 103 healthy controls (61 females, mean age = 31.88) were selected from two multicenter studies on the neuro-genetic causes of major depression, schizophrenia, and bipolar disorder (Erk et al., 2014; Wackerhagen et al., 2019). First-degree relatives were included in this study if they had at least one first-degree relative diagnosed with MDD. Except for family history of MDD in first-degree relatives, neither first-degree relatives nor healthy controls had a personal or familial history of lifetime axis I disorders. Inclusion criteria for patients were a current diagnosis of a recurrent depressive disorder or a depressive episode that was either severe or had lasted for at least 2 years. Psychiatric history was confirmed using the German version of the Structured Clinical Interview for DSM-IV-TR Axis I Disorders (Wittchen, Zaudig, & Fydrich, 1997). Thirty-three of the patients were receiving psychotropic medication at the time of the functional magnetic resonance imaging (fMRI) investigation (online Supplementary Table S1). For further details of the study cohorts, see Wackerhagen et al. (2019).

The study was approved by local ethics committees of the study sites. All participants provided written informed consent.

### Psychological measurements

The current level of negative affectivity was assessed using the Beck Depression Inventory (Hautzinger, Bailer, Worall, & Keller, 1994), the depression scale of the Symptom Checklist-90 (SCL-90-R; Derogatis, 1977), State-Trait Anxiety Inventory-State (STAI-S; Spielberger, Gorsuch, & Lushene, 1970), State-Trait Anxiety Inventory-Trait (STAI-T; Spielberger et al., 1970), and the neuroticism scale of the NEO-Five Factor Inventory (NEO-FFI; Costa & McCrae, 1992). A composite score of negative affect was computed for each participant based on BDI, SCL-90 Depression, STAI-T, and NEO-FFI Neuroticism scales using principal component analysis (see online Supplementary material S3 for details of principal component analysis).

### Experimental paradigm

We used a block-designed fMRI task adapted from Hariri et al. (2002) to investigate the neural correlates of implicit emotion processing. The task had two conditions: face-matching and shape-matching (online Supplementary Fig. S2). During the face-matching condition, participants were asked to match one of the two simultaneously presented faces (angry or fearful faces) with the identical target face. During the sensorimotor control condition, participants matched geometric shapes similarly. Eight blocks (four blocks for each condition) were presented in alternating order. Each block consisted of six trials of 5 s and started with a brief instruction. The total task duration was 256 s.

### Image acquisition and preprocessing

The images were acquired on a Siemens Magnetom Trio (Siemens, Erlangen, Germany) 3T MRI scanner using identical scanning protocols. During the task, 134 functional images were obtained using an asymmetric gradient echo-planar sequence sensitive to blood oxygen level-dependent (BOLD) contrast (28 slices, TE = 30 ms, TR = 2000 ms, flip angle = 80°, FoV = 192 mm, voxel size = 3 × 3 × 4 mm).

Image preprocessing was performed using SPM12 (http://www.fil.ion.ucl.ac.uk/spm/) and included slice timing correction, motion correction, structural and functional image co-registration, segmentation, normalization to the Montreal Neurological Institute (MNI) 152 template, and smoothing using a kernel with a full-width half-maximum of 8 mm. Furthermore, to quantify mean head motion for each participant, we computed frame-wise displacement based on rigid body transformation parameters (Power et al., 2014). For a detailed description of image acquisition and preprocessing, see online Supplementary material S5.

### Generalized linear modeling

Generalized linear modeling (GLM) implemented in SPM12 was performed to estimate brain responses. Each experimental condition (face-matching and shape-matching) and instructions were convolved with a canonical hemodynamic response function. Six motion parameters and time series from white matter and cerebrospinal fluid were entered into the subject-level analysis as nuisance covariates to correct for motion and physiological noise.

At the group level, a one-sample *t* test was performed to find brain regions showing the main effect of the task (faces > shapes). Additionally, we conducted one-way analysis of variance (ANOVA) to examine group differences in the main effect of the task. Age, sex, education, study site, and mean head motion were included in all analyses as covariates.

### Regions of interest selection

Based on our GLM results, we selected the following regions of interest (ROIs) to use in subsequent DCM analysis: fusiform gyrus, amygdala, anterior cingulate cortex, dorsolateral prefrontal cortex, orbitofrontal cortex, and insula (see online Supplementary Table S3 for the MNI coordinates). All ROIs were chosen bilaterally, except the anterior cingulate cortex. The model included 11 ROIs in total.

To account for individual differences in the peak locations of brain activation, we searched for the local maxima nearest to the group-level coordinates within anatomical boundaries of given ROIs (thresholded at *p* < 0.05, uncorrected). Regional responses were then summarized with the first-eigenvariate of all activated voxels within a 6 mm sphere of the subject-specific local maxima. For participants showing no experimental effect within a given ROI, the first eigenvariate of time series was extracted from a 6 mm sphere of the group-level maximum (Zhou et al., 2018). Participants who did not show consistent experimental effect for more than two ROIs within a hemisphere were excluded from further analysis (*n* = 13). For a detailed description, see online Supplementary material S6.

### Dynamic causal modeling

Effective connectivity was investigated using DCM for fMRI (DCM 12.5, Revision 7479). DCM is a Bayesian framework that uses the multiple input-state-output model to make inferences about hidden neural states underlying measured time series. The inputs correspond to the stimulus function which can elicit changes in neural activity. The states represent neural responses and other neurophysiological variables, whereas the outputs are region-specific BOLD responses (Friston et al., 2003). To generate time series from underlying causes (e.g. neural fluctuations and connection strengths), DCM forward model combines a neuronal model with a hemodynamic model. Since the predicted time series by DCM is dependent on neuronal model parameters (e.g. connectivity architecture), DCM aims to find the model which explains the data best, corresponding the model with a minimal discrepancy between predicted time series and observed time series (Friston et al., 2003; for a detailed description, see online Supplementary material S7).

In this study, we wanted to investigate the effect of viewing emotional faces on intrinsic connections. For this purpose, we specified a fully connected model with 121 neural coupling parameters (see online Supplementary Fig. S5), which allowed us to compare all possible nested models within the network. The driving input (i.e. emotional faces) entered the model through bilateral fusiform gyri and propagated through the network via intrinsic connections. After the model estimation, we performed diagnostics to check the quality of the DCM model fitting to ensure that model inversion was successful (Zeidman et al., 2019a). Participants whose explained variance by the model was less than 10% (*n* = 8) were excluded from further analyses. The final sample for group-level DCM analyses included 179 participants (see online Supplementary Fig. S6 for the flow diagram of study participants).

### Empirical Bayes for group DCM

We quantified commonalities and group differences in effective connectivity using parametric empirical Bayes (PEB). The PEB is a hierarchical Bayesian framework to estimate effective connectivity parameters at the group level. Here, we set three PEB analyses to compare three groups (e.g. controls *v.* patients, controls *v.* relatives, and relatives *v.* patients). Age, sex, education, study site, and mean head motion were included in all analyses as covariates (for a detailed description see online Supplementary material S9). Since PEB is a multivariate Bayesian GLM, in which all the connectivity parameters are fitted at once to optimize the model evidence, no correction for multiple comparisons is required in contrast to a frequentist approach.

After group differences in effective connectivity were determined, we used a similar framework as in Wackerhagen et al. (2019) to associate these group differences with disease pathology, risk, and resilience. For disease-related changes in effective connectivity, we looked at both ‘controls *v.* patients’ and ‘relatives *v.* patients’ contrasts and determined the connections which are altered in patients compared to both controls and relatives. In the same way, we looked at both ‘controls *v.* patients’ and ‘controls *v.* relatives’ contrasts to determine shared features by patients and relatives (i.e. risk-related changes). Finally, we looked at ‘controls *v.* relatives’ and ‘relatives *v.* patients’ contrasts and determined the connections which are unique in the unaffected familial risk group and might thus serve a compensatory function potentially promoting resilience.

### Brain–behavior relationship

We identified the associations between negative affect scores and effective connectivity during the face-matching task using the PEB framework. The analysis was performed separately for each group. We here focused only on the parameters associated with disease, risk, and resilience for the sake of simplicity. However, we reported the associations between negative affect and other effective connectivity parameters in online Supplementary Table S9. Positive associations indicated that the strength of an effective connection increases when negative affect increases, whereas negative associations indicated that the strength of an effective connection decreases when negative affect increases. Additionally, we tested whether the associations between negative affect and selected effective connectivity parameters differ between groups (Eid, Gollwitzer, & Schmitt, 2011).

### Effect of antidepressant treatment

Since the majority of the patients were under antidepressant treatment, we wanted to examine the potential effect of medication use on group differences in task-related activity and connectivity. For that purpose, we included a dummy variable representing the medication status in the second-level GLM and DCM analyses as a covariate of no interest in addition to the covariates above.

Unless otherwise stated, all second-level DCM results (i.e. group comparisons, brain–behavior relationship, and effect of antidepressant treatment) were reported based on the posterior probability (free energy with *v.* without parameter) greater than 0.95 criterion.

## Results

### Behavioral results

As shown in Table 1, groups did not differ in terms of age, sex, years of education, and task performance. However, there were significant group differences in study site and head motion. Patients had significantly higher head motion (Mdn = 0.12) than controls (Mdn = 0.09, *p* = 0.04) and relatives (Mdn = 0.08, *p* < 0.001). Moreover, as expected, the one-way ANOVA test revealed a significant main effect of group in all psychological measurements (for test statistics of post-hoc comparisons, see online Supplementary Table S5).

**Table 1.** Sample characteristics

	Controls (*N* = 103)	Relatives (*N* = 49)	Patients (*N* = 48)	df	*F* χ^2^	*p*
Demographics						
Sex, M/F, *N*	42/61	16/33	14/34	2	2.23	0.33
Age (years), *M* (s.d.)	31.25 (9.33)	28.49 (8.11)	31.88 (8.97)	197	2.10	0.12
Education (years), *M* (s.d.)	14.14 (2.89)	14.49 (2.80)	14.78 (2.19)	2	0.92	0.40
Study site, *N*				4	26.76	<0.001*
Bonn	13	16	0			
Mannheim	30	15	25			
Berlin	60	18	23			
Mean head motion, Mdn (IQR)	0.09 (0.05)	0.08 (0.04)	0.12 (0.10)	197	12.67	0.002*
Type of familial relationship, *N*						
Offspring/sibling/parent		35/12/1				
Task performance						
Reaction time (s), *M* (s.d.)						
Shapes	1.11 (0.21)	1.12 (0.29)	1.12 (0.19)	197	0.02	0.98
Faces	1.21 (0.24)	1.25 (0.30)	1.19 (0.19)	197	0.74	0.48
Accuracy, *n* (%)						
Shapes	23.18 (96.58)	23.32 (97.21)	23.27 (96.96)	196	0.22	0.80
Faces	23.64 (98.5)	23.67 (98.62)	23.75 (98.96)	196	0.19	0.83
Psychological measurements						
SCL-90 depression, Mdn (IQR)	0.08 (0.29)	0.08 (0.27)	1.54 (1.27)	197	88.26	<0.001*
BDI, Mdn (IQR)	1 (3.5)	3 (4.5)	21 (16.5)	197	99.78	<0.001*
STAI-S, *M* (s.d.)	31.56 (6.51)	31.85 (5.32)	49.72 (10.89)	162	89.88	<0.001*
STAI-T, *M* (s.d.)	33.46 (8.43)	35.76 (8.10)	56.53 (10.65)	191	112.32	<0.001*
NEO-FFI neuroticism, *M* (s.d.)	14.32 (6.62)	17.5 (6.99)	32.53 (8.04)	193	110.80	<0.001*
Negative affect, Mdn (IQR)	−0.56 (0.60)	−0.35 (0.73)	1.59 (0.98)	191	52.95	<0.001*

BDI, Beck's Depression Inventory; HDRS, Hamilton Depression Rating Scale; NEO, NEO-Five Factory Inventory; SCL90-R Depression, Symptom Checklist 90 Revised Depression Scale; STAI-S, State Trait Anxiety Inventory – State Anxiety; STAI-T, State Trait Anxiety Inventory – Trait Anxiety.

*p < 0.05.

### Task-related brain activity

**Fig. 1.**
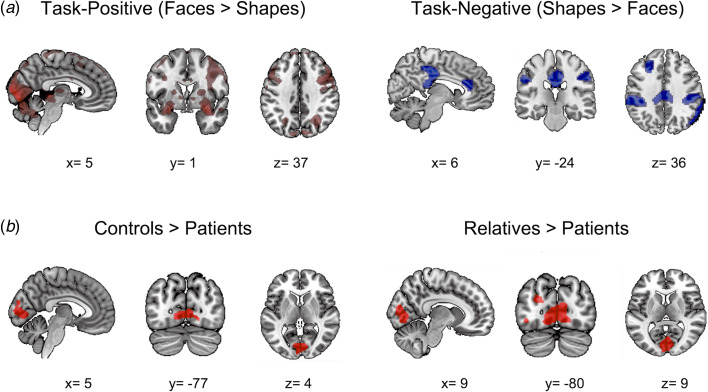
Task-related brain activity across and between groups. The upper panel (*a*) shows brain regions exhibiting increased (left panel, red color) and decreased (right panel, blue color) activation during the face-matching condition compared to the shape-matching condition (*p* < 0.05; whole-brain FWE-corrected). The lower panel (*b*) shows group differences in the task-related activation in healthy controls (left panel) and first-degree relatives (right panel) compared to patients with MDD. Here, red color indicates increased regional responses during the face-matching condition relative to the shape-matching condition in the respective group comparison.

Across participants, the face-matching task elicited more activation than the shape-matching task in the visual cortex, fusiform gyrus, dorsal prefrontal cortex, subcortical areas (thalamus, amygdala, hippocampus, putamen), and cerebellum (*p* < 0.05, family-wise error (FWE) corrected; Fig. 1 and online Supplementary Table S6). The shape-matching task elicited more activation than the face-matching task in bilateral parietal lobes, middle and anterior cingulate cortex, middle occipital cortex, and middle frontal gyrus (*p* < 0.05, FWE-corrected).

The one-way ANOVA test showed a significant effect of group on brain responses (faces > shapes). Post-hoc group comparisons revealed that patients exhibited significantly decreased activation in visual cortex (bilateral lingual gyrus, superior occipital gyrus, and calcarine sulcus) compared to controls and relatives (*p* < 0.05, FWE-corrected; Fig. 1 and online Supplementary Table S7). We did not observe any significant group difference between controls and relatives in task-related brain activation (*p* < 0.05, FWE-corrected).

### Effective connectivity

Between-group differences in effective connectivity during face-matching condition are listed in online Supplementary Table S8. We reported only the connection parameters with a probability greater than 95% (posterior probability >0.95), which corresponds to strong evidence. Having determined between-group differences, we identified connection parameters associated with depressive state, risk, and resilience for MDD.

**Fig. 2.**
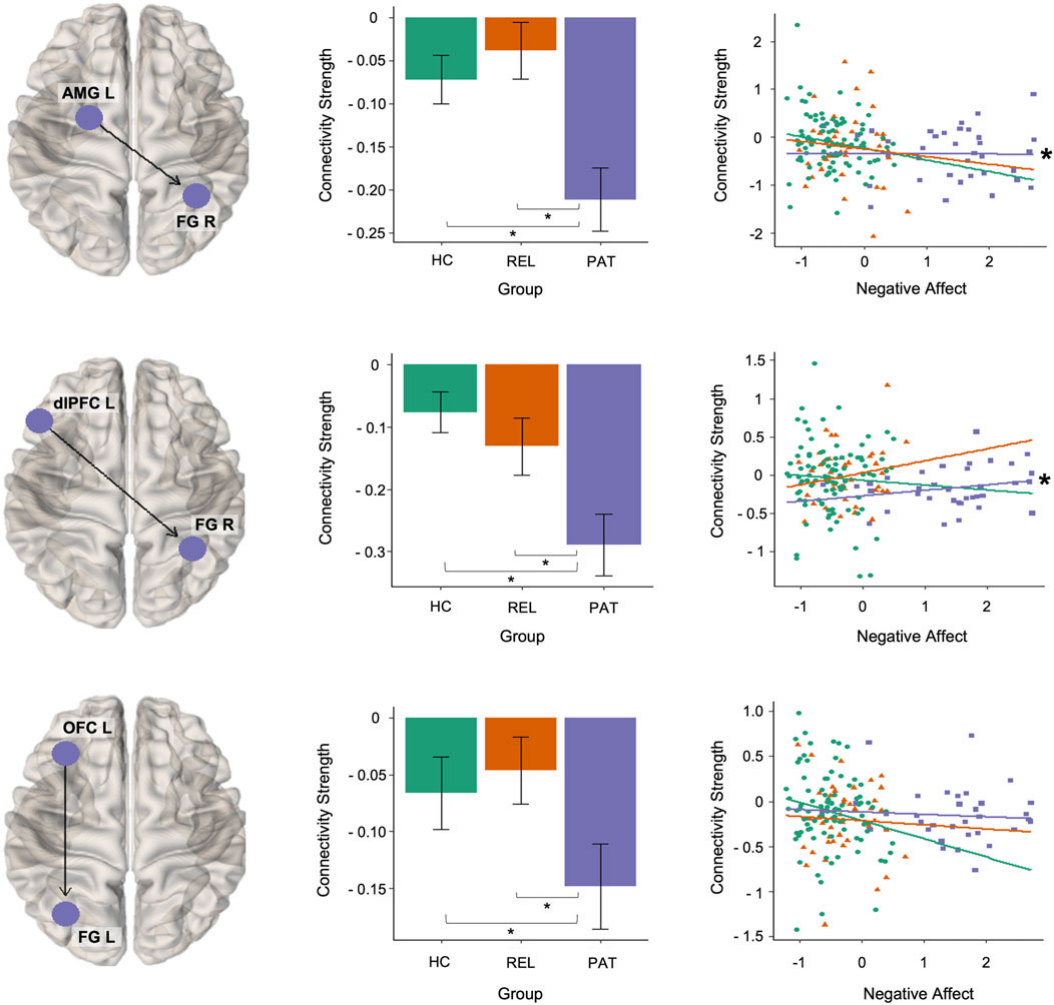
Disease-related alterations in effective connectivity. The middle panel shows the group means of the effective connection parameters associated with depressive pathology. Error bars indicate 95% Bayesian confidence interval. Group differences with strong evidence [i.e. posterior probability (free energy with *v.* without parameter) are larger than 0.95] are marked with an asterisk. Negative affect scores and estimated posterior means of connection parameters are plotted in the right panel for visualization. The associations with strong evidence are marked with an asterisk. HC, healthy controls; PAT, patients with major depression; REL, first-degree relatives of patients with depression.

Disease state was associated with lower effective connectivity from left amygdala and left dorsolateral prefrontal cortex to right fusiform gyrus and from left orbitofrontal cortex to left fusiform gyrus (Fig. 2). As seen in Fig. 2, the estimated group means for these intrinsic connection parameters had negative values. That is, high activity in the source region leads to a decrease in activity in the target region (i.e. inhibitory influence). Thus, lower effective connectivity observed in MDD patients corresponds to more inhibitory influence from amygdala and frontal regions to fusiform gyrus.

**Fig. 3.**
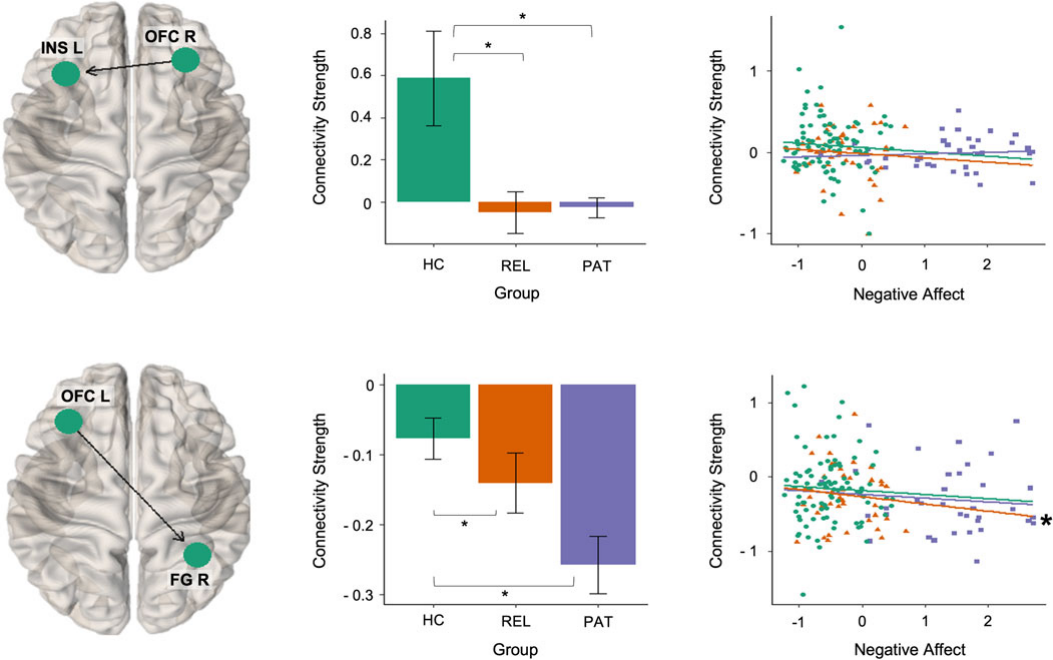
Risk-related alterations in effective connectivity. The middle panel shows the group means of the effective connection parameters associated with risk for depression. Error bars indicate 95% Bayesian confidence interval. Group differences with strong evidence are marked with an asterisk. Negative affect scores and estimated posterior means of connection parameters are plotted in the right panel for visualization. Associations with strong evidence are marked with an asterisk. HC, healthy controls; PAT, patients with major depression; REL, first-degree relatives of patients with depression.

Risk for MDD was associated with decreased effective connectivity from right orbitofrontal cortex to left insula and from the left orbitofrontal cortex to right fusiform gyrus (Fig. 3). The connectivity from right orbitofrontal cortex to left insula was positive (i.e. excitatory) in controls, whereas this connection was absent in relatives and patients. For the connectivity from the left orbitofrontal cortex to right fusiform gyrus, all groups exhibited inhibitory signaling. Thus, decreased connectivity in relatives and patients indicated more inhibitory influence. Moreover, this alteration was gradual (patients < relatives < controls).

**Fig. 4.**
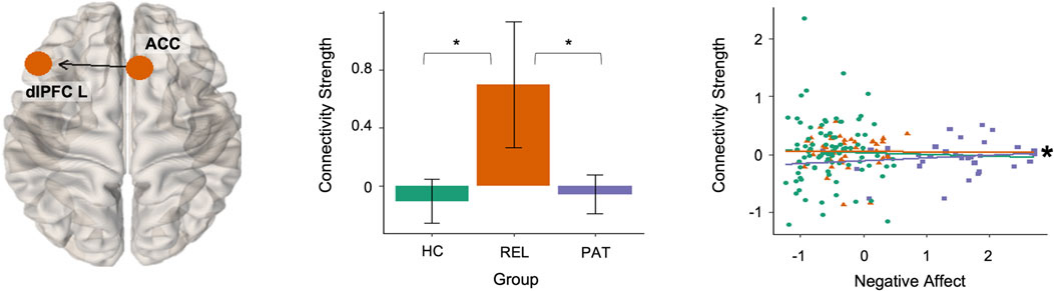
Resilience-related alterations in effective connectivity. The middle panel shows the group means of the effective connection parameter associated with resilience. Error bars indicate 95% Bayesian confidence interval. Group differences with strong evidence are marked with asterisk. Negative affect scores and estimated posterior means of connection parameters are plotted in the right panel for visualization. Associations with strong evidence are marked with an asterisk. HC, healthy controls; PAT, patients with major depression; REL, first-degree relatives of patients with depression.

Effective connectivity from anterior cingulate cortex to left dorsolateral prefrontal cortex was elevated in relatives compared to controls and patients in the resilience contrast (Fig. 4). The estimated values for this connection were positive for relatives, reflecting excitatory signaling, whereas the estimated values were around zero for patients and controls.

### Brain–behavior relationship

The analysis of the brain–behavior relationship revealed that effective connectivity from left amygdala to right fusiform gyrus (*β* coefficient = −0.08) and from left orbitofrontal cortex to right fusiform gyrus (*β* coefficient = −0.11) were negatively associated with negative affect scores in MDD patients. In contrast, we identified positive associations between negative affect and effective connectivity from left dorsolateral prefrontal cortex to right fusiform gyrus (*β* coefficient = 0.08) and from anterior cingulate cortex to left dorsolateral prefrontal cortex (*β* coefficient = 0.13) in MDD patients. We found no association between negative affect and effective connectivity parameters in controls and relatives. Moreover, MDD patients (*r*_s_ = 0.28) differed significantly from controls (*r*_s_ = −0.09, *z* = 1.86, *p* < 0.05) and at a trend level from relatives (*r*_s_ = −0.08, *z* = 1.57, *p* = 0.06) in terms of the association between negative affect and the connectivity from anterior cingulate cortex to left dorsolateral prefrontal cortex.

### Effect of antidepressant treatment

When the mediation status was considered, group differences in task-related activity remained the same. However, antidepressant treatment had an influence on effective connectivity parameters associated with the disease state. When we included the medication status as a covariate of no-interest, we found that the previously identified disease-related alterations in effective connectivity (more inhibitory influences from the amygdala and lateral prefrontal cortex to fusiform gyrus) were no longer present. When we looked at the effect of antidepressant treatment on effective connectivity, we found that these effective connections were negatively associated with medication use (see online Supplementary Fig. S8). That is, more inhibitory influence from the amygdala and lateral prefrontal cortex to fusiform gyrus was associated with medication use.

Potential risk and resilience markers identified in the current study remained unchanged when the effect of antidepressant use was considered. Moreover, we identified two further alterations in connectivity: increased connectivity from left amygdala to left insula in relatives and patients compared to controls as a potential marker of risk and increased connectivity from left insula to right fusiform gyrus in relatives compared to controls and patients as a potential marker of resilience.

## Discussion

The present study investigated brain responses and effective connectivity during the face-matching task in patients with MDD, unaffected first-degree relatives, and healthy participants. Similar to our previous study (Wackerhagen et al., 2019), the current study aimed to test the limbic-cortical imbalance hypothesis in relation to pathology, risk, and resilience for MDD. In the current study, MDD pathology was associated with decreased effective connectivity from the left amygdala and left lateral cortex to the fusiform gyrus. This might indicate a reduced limbic-cortical integration as well as an altered top-down control of higher visual regions in the depressive state. However, different from the previous study (Wackerhagen et al., 2019), we did not identify any alteration in amygdala-prefrontal cortex connectivity as a disease, risk, or resilience marker. Nevertheless, these results are not necessarily contradictory since our DCM model did not include those middle frontal regions whose connectivity with amygdala was found to be related to pathology and resilience in the previous study.

Moreover, we previously found decreased amygdala connectivity with fusiform gyrus as a marker of risk for MDD (Wackerhagen et al., 2019). However, the identified cluster in right fusiform gyrus was relatively small (*k* = 10, *p* < 0.001). Here, we also found altered connectivity from amygdala to right fusiform gyrus but it represented a disease marker and did not survive when medication status was included as a covariate. Instead, we identified decreased connectivity from the right orbitofrontal cortex to the left insula and from the left orbitofrontal cortex to the right fusiform gyrus as risk markers. Taken together, a reduced stimulus-dependent integration of visual regions was identified as potential risk mechanisms in both studies. In addition, the same previous study identified increased superior frontal cortex connectivity with anterior cingulate cortex (*k* = 132, *p* < 0.05, FWE-corrected) in an exploratory analysis. The current study similarly found increased connectivity from anterior cingulate cortex to dorsolateral prefrontal cortex as a marker of resilience, suggesting a potential compensatory mechanism in the cognitive network. These findings may indicate that the results are more likely to be replicated with DCM when more subtle effects existed.

It is important to note that comparing the results from PPI and DCM can be challenging since these methods are based on different statistical approaches (classical *v.* Bayesian inference), and results are sensitive to ROI selection and model space. However, both methods are useful and offer different perspectives related to the research question. Here, we aimed to allow for new insights into the neural model of MDD by using a model with brain regions that play an important role in emotion processing and MDD pathology, and our DCM results showed that beyond the amygdala, interactions between several cortical regions can be important to understand the disease, risk, and resilience brain alterations in MDD.

### Disease state

Compared to relatives and controls, MDD patients exhibited lower activity in several visual areas and lower effective connectivity (i.e. more inhibitory influence) from higher-order areas to fusiform gyrus during the face-matching task.

Specifically, MDD patients showed more inhibitory influence from the left amygdala to right fusiform gyrus than controls and relatives. This increased inhibitory influence was also associated with higher negative affect in MDD patients. Several studies showed that the amygdala has a modulatory role over the visual cortex during emotional face processing (Das et al., 2005; Furl, Henson, Friston, & Calder, 2013; Morris et al., 1998; Vuilleumier, Armony, Driver, & Dolan, 2001; Vuilleumier, Richardson, Armony, Driver, & Dolan, 2004; Williams, 2006). Via the modulatory connections, the amygdala can enhance or diminish the sensory representation of a stimulus in visual regions (Pessoa & Adolphs, 2010). Thus, these findings indicate that the amygdala detects the motivational significance of a stimulus and then modulates the activity of visual regions by increasing or decreasing visual attention to the stimulus based on its motivational significance. Since the fusiform gyrus has a crucial role in face processing and social perception (Bickart, Dickerson, & Barrett, 2014; Fairhall & Ishai, 2007; Fisher, Towler, & Eimer, 2016; Haxby, Hoffman, & Gobbini, 2000, 2002), excessive inhibition of fusiform gyrus activity by the amygdala can cause diminished visual attention to motivationally important stimuli in MDD patients.

Additionally, disease pathology was associated with increased inhibitory influence from the lateral prefrontal regions to fusiform gyrus. MDD patients with higher negative affect also exerted less inhibitory influence from left dorsolateral prefrontal cortex to right fusiform gyrus. These results are compatible with previous studies reporting decreased functional connectivity between frontal and visual regions in MDD patients during an emotional task (Frodl et al., 2010; Tak et al., 2021) and at resting state (Chen et al., 2019; Teng et al., 2018). The lateral prefrontal cortex plays an important role in the integration of cognitive and emotional information (Erk, Kleczar, & Walter, 2007; Gray, Braver, & Raichle, 2002) and inhibition of task-irrelevant stimuli (Dolcos, Kragel, Wang, & McCarthy, 2006; García-Pacios, Garcés, Del Río, & Maestú, 2015; Wessa, Heissler, Schönfelder, & Kanske, 2013). Moreover, similar to the amygdala, it can modulate the activation of sensory and association cortices (Hooker & Knight, 2010; Notzon, Steinberg, Zwanzger, & Junghöfer, 2018). Previous studies showed that emotional stimulus can interfere goal-directed behavior (Dolcos et al., 2006), and top-down control of emotional distractors can result in better task performance (Minamoto, Osaka, & Osaka, 2010; Ziaei, Peira, & Persson, 2014). Therefore, more inhibitory influence detected in patients could be related to inhibition of emotional distractors (e.g. facial expression) during the identity matching and contribute to maintaining the same level of task performance with controls and relatives by preventing the disruptive effect of emotional distractors on working memory (Minamoto et al., 2010). However, due to the lack of variability in task performance, we could not test this hypothesis. Moreover, the utilized task is not cognitively challenging. Therefore, the alleged compensatory mechanism cannot be generalized to cognitively demanding tasks since MDD patients may fail to inhibit emotional distractors when task difficulty increases.

It is important to note that these alterations did not survive when the effect of antidepressant treatment was considered. These results are in line with previous studies showing that antidepressant treatment alters the connectivity during emotional face processing in MDD patients (Chen et al., 2008; Vai et al., 2016). Here, we found that backward connections from higher-order regions to fusiform gyrus became more negative (i.e. more inhibitory) with the antidepressant treatment. Importantly, medicated and non-medicated patients did not differ in terms of BDI scores. These results indicate that antidepressant treatment can have an important influence on effective connectivity patterns even in the absence of behavioral differences. Thus, its effect on brain connectivity must be considered in future studies.

### Potential risk markers

We found that the absence of effective connectivity from right orbitofrontal cortex to the left insula was associated with risk for MDD. The insula has been related to affective processing and has bidirectional connections with orbitofrontal cortex (Gasquoine, 2014). The increased coupling between the insula and prefrontal cortex in healthy controls was linked to attenuated distraction (Pedale, Macaluso, & Santangelo, 2019) and decreased negative emotion during suppression (Goldin, McRae, Ramel, & Gross, 2008). Thus, the positive fronto-insular coupling in controls may reflect a functional mechanism, possibly suppression of irrelevant information, which helps participants to deal with emotional distractors, whereas the absence of this connection in patients and relatives might reflect a failure to allocate a functional mechanism which can help to decrease negative emotion.

Furthermore, similar to MDD patients, relatives exhibited decreased (more inhibitory) connectivity from left orbitofrontal cortex to right fusiform gyrus compared to controls. A recent study reported that the main effect of familial risk for depression (i.e. patients and healthy controls with *v.* without a family history of MDD) was associated with altered functional connectivity between the orbitofrontal cortex and visual regions (Opel et al., 2017). Although we did not directly investigate the main effect of familial risk in this study in the same way, our results indicate that orbitofrontal cortex might be an important brain region to investigate risk-related changes in depression.

Contrary to our hypothesis, we did not identify decreased effective connectivity between the amygdala and frontal regions as a putative risk marker for depression. In line with previous studies (Chen et al., 2008; Dannlowski et al., 2009; Erk et al., 2010; Fales et al., 2008; Lu et al., 2012), MDD patients showed decreased effective connectivity from left dorsolateral prefrontal cortex to left amygdala compared to controls. However, relatives did not differ from either controls or patients given the strong evidence (posterior probability >0.95). Although evidence from separate studies support that both MDD patients and individuals at familial risk for MDD exhibit altered amygdala connectivity with the prefrontal cortex, the precise location of the coupling region within the prefrontal cortex may differ according to the groups. Since our DCM included only lateral prefrontal regions (BA 46 and 47) due to their prominent activation during the face-matching task, other prefrontal regions which are not included in the model, such as medial prefrontal regions (Wackerhagen et al., 2019, 2017), may play a more important role in risk for MDD.

### Potential resilience markers

During the face-matching task, relatives exhibited increased excitatory effective connectivity from anterior cingulate cortex to left dorsolateral prefrontal cortex, whereas this connection was absent in controls and patients. Increased functional connectivity between the anterior cingulate cortex and left superior frontal gyrus in first-degree relatives was also observed in our previous PPI study and was interpreted as a potential marker of resilience (Wackerhagen et al., 2019). The anterior cingulate cortex is responsible for various aspects of emotional processing (Etkin, Egner, & Kalisch, 2011; Etkin, Egner, Peraza, Kandel, & Hirsch, 2006) and constitutes the cognitive-control network together with the dorsolateral prefrontal cortex (Li et al., 2018). Previous studies have linked the neural coupling between the anterior cingulate cortex and the dorsolateral prefrontal cortex to a better performance in attention shifting (Kondo, Osaka, & Osaka, 2004) and increased top-down attentional control (Comte et al., 2016) in healthy participants. Thus, enhanced fronto-cingulate connectivity in relatives can serve as a resilience capacity mechanism by providing more cognitive control during affective face processing.

However, enhanced connectivity from the anterior cingulate cortex to the dorsolateral prefrontal cortex was related to increased negative affect in MDD patients. Although previous studies associated enhanced fronto-cingulate connectivity with positive outcomes in healthy participants, a recent study showed that weaker connectivity between the anterior cingulate cortex and the dorsolateral prefrontal cortex was beneficial for depression recovery in MDD patients before and after 8 weeks of antidepressant treatment (Meyer et al., 2019).

Furthermore, it is important to note that we here attempted to identify resilience-related changes in effective connectivity using a cross-sectional design. Although it brings some practical advantages, it is challenging to deem a unique feature as a resilience marker, since first-degree relatives still have the possibility of developing depression in the future given the relatively wide age of onset range of this disorder (Kessler et al., 2005). Therefore, the observed alteration in first-degree relatives can also reflect a risk marker that occurs before the onset of the disease (Wackerhagen et al., 2019).

### Limitations

Our findings need to be interpreted in light of some limitations. First, we assume that our experimental design measures neural responses to implicit emotional processing since all faces embodied expressions of emotion. However, the utilization of geometrical shapes in the control condition instead of neutral faces made it impossible to dissociate the effect of emotional valence from basic face processing. Thus, we could not investigate the direct effect of emotional valence on brain activity and connectivity in this study.

Second, the current concept of resilience (Kalisch et al., 2017) requires that measures of stressor-load should be put into relation with symptomatology across time to assess resilience as an outcome. Therefore, the potential protective mechanisms found in our group comparisons and their relevance for resilient stress-coping needs to be further evaluated, preferably in longitudinal studies.

Lastly, we here used DCM as a causal search paradigm by specifying a fully connected model. Connection parameters that did not contribute to model evidence were then pruned away from the model. This procedure uses a greedy search algorithm that finds the best solution at each step rather than comparing all possible nested models. When the number of ROIs in the model increased, model space grows exponentially, and using a greedy search algorithm with a large model space can introduce bias to construct post-hoc explanations for the surviving parameters (Zeidman et al., 2019b).

## Conclusion

Our results suggest that the depressive state alters top-down control of higher visual regions, which are important for emotional face processing. Decreases in effective connectivity from the orbitofrontal cortex to insula and fusiform gyrus present putative risk markers. Enhanced connectivity within the cognitive control network was a distinguishing characteristic in relatives, whose etiological implications need to be further investigated in longitudinal studies. If further research supports these hypotheses, brain regions whose activity and connectivity pattern are related to the depressive state, risk, and resilience can be used as targets in novel treatments of depression, such as neurofeedback (Koush et al., 2013; Linhartová et al., 2019; Paret et al., 2016; Young et al., 2018).

## Supplementary material

For supplementary material accompanying this paper visit https://doi.org/10.1017/S0033291722000824. click here to view supplementary material
