# Supplementary material for: Effective connectivity during face processing in major depression – distinguishing markers of pathology, risk, and resilience
Source: Psychol Med. 2022 Apr 8;53(9):4139–51. doi: 10.1017/S0033291722000824 (PMC10317809; doi:10.1017/S0033291722000824)
Supplement: Supplementary file 1 [file S0033291722000824sup001.docx]

**Supplementary Material to**

**Effective connectivity during faces processing in major depression – distinguishing markers of pathology, risk, and resilience**

Seda Sacu ^1, a^, Carolin Wackerhagen ^2, a^, Susanne Erk ^2^, Nina Romanczuk-Seiferth ^2^, Kristina Schwarz^3^, Janina I. Schweiger^3,^ Heike Tost ^3^, Andreas Meyer-Lindenberg ^3^, Andreas Heinz ^2^, Adeel Razi ^4,5, b^, Henrik Walter ^1,2, b^

^1^ Berlin School of Mind and Brain, Humboldt Universität zu Berlin, Germany

^2^ Division of Mind and Brain Research, Department of Psychiatry and Psychotherapy CCM, Charité - Universitätsmedizin Berlin, corporate member of Freie Universität Berlin, Humboldt-Universität zu Berlin, and Berlin Institute of Health, Berlin, Germany

^3^ Department of Psychiatry and Psychotherapy, Central Institute of Mental Health, Mannheim, Germany

^4^ Wellcome Centre for Human Neuroimaging, Institute of Neurology, University College London, United Kingdom

^5^ Turner Institute for Brain and Mental Health & Monash Biomedical Imaging, Monash University, Australia

^a^ joint first authors ^b^ joint senior authors

Contents

[Background 2](#_Toc95233836)

[S1. The Theoretical Framework to Interpret Group Differences 2](#_Toc95233837)

[Methods 3](#_Toc95233838)

[S2. Clinical Characteristics 3](#_Toc95233839)

[S3. Principal Component Analysis 4](#_Toc95233840)

[S4. Experimental Paradigm 5](#_Toc95233841)

[S5. Image Acquisition and Preprocessing 6](#_Toc95233842)

[S6. Regions of Interest Selection 7](#_Toc95233843)

[S7. Dynamic Causal Modelling 10](#_Toc95233844)

[S8. Flow diagram 14](#_Toc95233845)

[S9. Empirical Bayes for Group DCM 15](#_Toc95233846)

[S10. DCM Pipeline 17](#_Toc95233847)

[Results 19](#_Toc95233848)

[S11. Post-hoc Tests for Psychological Measurements 19](#_Toc95233849)

[S12. Task-Related Brain Activity 20](#_Toc95233850)

[S13. Group Differences in Effective Connectivity 22](#_Toc95233851)

[S14. Medication Effect 23](#_Toc95233852)

[S15. Brain-Behavior Relationship 24](#_Toc95233853)

[References 26](#_Toc95233854)

# Background

## S1. The Theoretical Framework to Interpret Group Differences

**
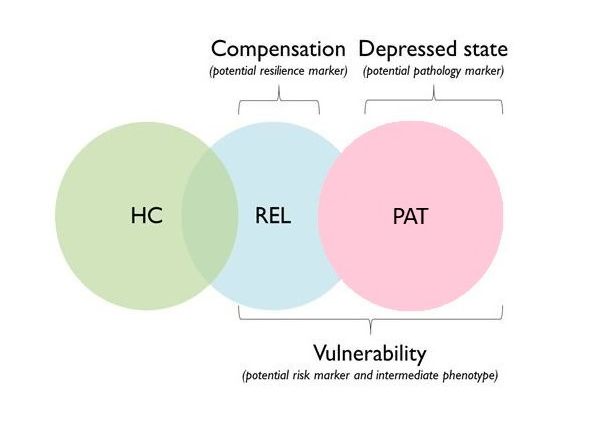
**

**Figure S1. The Theoretical framework for the interpretation of group differences.** According to the suggested framework (Wackerhagen et al., 2019), features that are unique to patients with major depressive disorder (MDD) compared to first-degree relatives and healthy controls represent depressed state/disease pathology (i.e., PAT≠REL=HC), whereas shared features by patients with MDD and first-degree relatives compared to healthy controls (i.e., PAT= REL≠ HC) and intermediate effects (PAT < REL < HC) constitute risk factors for MDD. Furthermore, specific features in first-degree relatives compared to patients with MDD and healthy controls can be interpreted as resilience capacity (i.e., REL≠ HC=PAT). Reprinted from “Amygdala Functional Connectivity in Major Depression – Disentangling Markers of Pathology, Risk and Resilience (Supplemental Information, *p*. 2)” by C. Wackerhagen, 2019, *Psychological Medicine*. Reprinted with permission. Abbreviations: HC, healthy controls; PAT, patients with major depressive disorder; REL, first-degree relatives of patients with MDD.

# Methods

## S2. Clinical Characteristics

**Table S1. Clinical characteristics of patients with major depressive disorder.**

| **Clinical Characteristics** |  |
| --- | --- |
| ICD-10 Diagnosis, n |  |
| F32. Depressive Episode | 9 |
| F33. Recurrent Depressive Disorder | 39 |
| HDRS, M (SD) | 13.68 (7.04) |
| CGI, M (SD) | 3.87 (1.3) |
| GAF, M (SD) | 62.28 (14.43) |
| Disease Severity, n |  |
| Mild | 7 |
| Moderate | 22 |
| Severe | 19 |
| Psychotropic Medication, n (%) | 33 (69) |
| Number of previous major depressive episodes in F33, Mdn (IQR) | 3 (2) [1-48] |
| First-degree relatives with major depressive disorder, n | 23 |
| Life-time Axis-I Comorbidity (ICD-10 code) |  |
| Alcohol abuse (F10) | 2 |
| Anxiety disorders (F4) | 11 |
| Eating disorders (F50) | 3 |
| None | 32 |

Abbreviations: CGI, Clinical Global Impression; GAF, Global Assessment of Functioning; HDRS, Hamilton Depression Rating Scale; ICD-10, International Statistical Classification of Diseases and Related Health Problems, 10th version.

## S3. Principal Component Analysis

Principal Component Analysis (PCA) is a statistical technique which is used to reduce the dimensionality of a large set of interrelated variables while retaining most of the information (Karamizadeh et al., 2013). Similar to our previous studies (Wackerhagen et al., 2017, 2019), we computed a negative affect composite score from four psychological tests (BDI, SCL-90 Depression, STAI-T and NEO-FFI Neuroticism) using PCA implemented in SPSS (SPSS Inc., Chicago, Illinois, USA). STAI-S scores were not included in the PCA because they were not obtained at the study site Mannheim. Due to the missing data, the sample size slightly decreased (N=191; 99 healthy controls, 46 first-degree relatives, and 46 MDD patients). The data was suitable for principal component analysis. Kaiser-Meyer-Olkin value exceeded 0.6, and p-value for Barlett's test of sphericity was smaller than 0.05. Inter-variable correlation ranged between r= 0.75 and 0.91. Only one component with an eigenvalue > 1 was found. The component was extracted using direct oblimin rotation method. The total variance explained by the component was 85.84%. The correlation between the component and each scale ranged between r= 0.92 and 0.94 (Table S2). A composite score was obtained using the regression method for each participant. The composite scores were then used to explore the relationship between negative affect and task-related effective connectivity.

**Table S2. Correlation between negative affect component score and psychological measurements.**

|  | **BDI** | **SCL**  **Depression** | **STAI-T** | **NEO-FFI**  **Neuroticism** |
| --- | --- | --- | --- | --- |
| Negative Affect r  (N=191) p | .93  <.001 | .94  <.001 | .93  <.001 | .93  <.001 |

Abbreviations: BDI, Beck Depression Inventory; NEO-FFI, NEO Five Factor Inventory; SCL, Symptom Checklist; STAI-T, State Trait Anxiety Inventory – Trait.

## S4. Experimental Paradigm

**
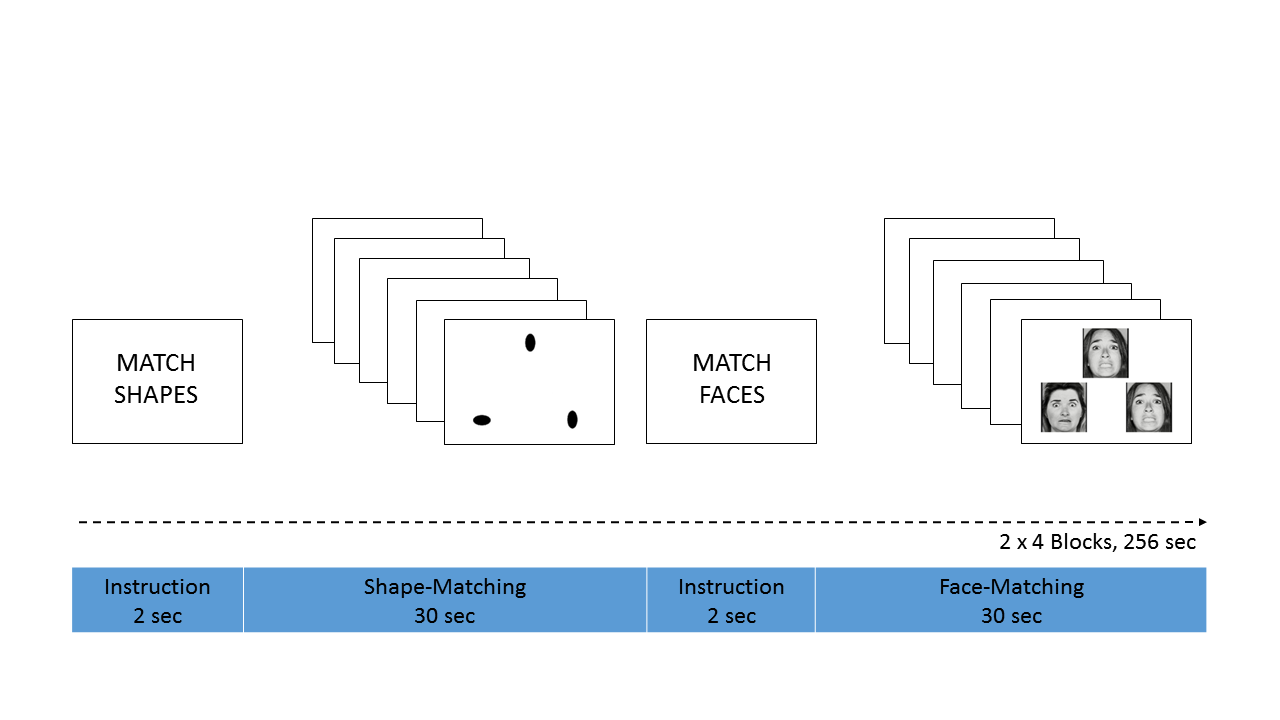
**

**Figure S2. Experimental Paradigm.** Each block started with a brief instruction. During the shape-matching blocks, participants matched one of the two simultaneously presented geometrical stimuli (bottom) with the identical target geometrical stimulus (top). During the face-matching blocks, participants matched one of the two simultaneously presented facial stimuli (bottom) with the identical target facial stimulus (top). Four blocks of shape-matching task interleaved with four blocks of face-matching task.

## S5. Image Acquisition and Preprocessing

The data were acquired on a Siemens Magnetom Trio (Siemens, Erlangen, Germany) 3T MRI scanner. Structural images were obtained using a sagittal magnetization-prepared rapid gradient echo (MP-RAGE) three-dimensional T1-weighted sequence (TE = 2.75 ms, TR = 1570 ms, flip angle = 15°, FoV = 256 mm, voxel size= 1×1×1 mm). Functional images were obtained using an asymmetric gradient echo-planar sequence sensitive to blood oxygen level-dependent (BOLD) contrast (TE= 30 ms, TR = 2000 ms, flip angle = 80°, FoV = 192 mm, voxel size = 3 × 3 × 4 mm). Whole-brain coverage for the functional data was obtained using 28 sequential 4 mm transversal slices, acquired parallel to the plane transecting the anterior and posterior commissure (AC-PC plane). In total, 134 volumes were acquired during the fMRI scan. A field inhomogeneity map was also obtained for the purpose of reducing spatial distortion in functional images (28 slices, TR= 400 ms, TE 1= 5.19 ms, TE 2= 7.65 ms, flip angle = 60°, FoV = 192 mm, voxel size= 3×3×4 mm).

All of the preprocessing steps were performed using the Statistical Parametric Mapping (SPM12 program (http://www.fil.ion.ucl.ac.uk/spm/). Slice acquisition-dependent time shifts were corrected per volume. All volumes were realigned to the first volume using a six-parameter (rigid body) linear transformation and unwrapped using the subject-specific field map. The resulting images were spatially normalized to a standard brain template in the Montreal Neurological Institute (MNI) coordinate space. Data were resampled to 3-mm isotropic voxels and spatially smoothed using an 8-mm full-width half-maximum Gaussian kernel. Furthermore, we computed frame-wise displacement based on rigid body transformation parameters obtained after realignment to quantify mean head motion for each participant (Power et al., 2012, 2014; Zhou et al., 2018).

## S6. Regions of Interest Selection

Dynamic Causal Modelling (DCM) is a hypothesis-driven approach that is motivated by an experimental design (Friston et al., 2003). Since DCM aims to identify a possible neural circuit that explains brain activity induced by experimental stimuli, regions of interest (ROIs) for DCM analysis are generally selected based on brain activation during an experimental task (Zeidman et al., 2019a). We, therefore, chose brain regions that showed the main effect of the task (faces > shapes) across participants (p < .05; FWE-corrected; Table S3), which are also deemed as important for emotion processing and regulation. In addition to functional constraints, anatomical masks were used to make sure that regional time series were extracted within the boundaries of a given ROI (Table S4). We used SPM Anatomy Toolbox (Eickhoff et al., 2005) to create anatomical masks for basolateral amygdala and lateral fusiform gyrus. For the rest of the ROIs, we created anatomical masks using Automated Anatomical Labelling Atlas implemented in the Wake Forest University (WFU) PickAtlas toolbox (https://www.nitrc.org/projects/wfu_pickatlas/). For each participant, we first searched for the local maxima nearest to the group-level coordinates within anatomical boundaries of a given ROI (p < 0.05, uncorrected). Regional responses were then summarized with the first-eigenvariate of all activated voxels within 6 mm sphere of the subject-specific local maxima. If a participant failed to show an experimental effect within a given ROI, the first eigenvariate of time series was extracted from a 6 mm sphere of the group-level maximum (Zhou et al., 2018).

**Table S3. Anatomical Locations of Regions of Interest.**

| **Regions of Interest** | **MNI Coordinates** | **T** |
| --- | --- | --- |
| Fusiform Gyrus L | -36 -73 -13 | 29.69 |
| Fusiform Gyrus R | 39 -55 -16 | 28.29 |
| Amygdala L | -24 -4 -19 | 13.88 |
| Amygdala R | 27 -4 -19 | 14.55 |
| Anterior Cingulate Cortex | 9 26 20 | 7.00 |
| Dorsolateral Prefrontal Cortex L | -51 29 23 | 13.39 |
| Dorsolateral Prefrontal Cortex R | 54 32 20 | 17.59 |
| Orbitofrontal Cortex L | -36 32 -16 | 12.19 |
| Orbitofrontal Cortex R | 33 32 -16 | 14.73 |
| Insula L | -36 23 -1 | 8.10 |
| Insula R | 36 29 -1 | 9.00 |

Regions of Interest were located at group-level peak MNI coordinates of the faces > shapes contrast at a significance threshold of p < 0.05 (FWE-corrected).

**Table S4. The anatomical masks used for time series extraction.**

| **Regions** | **Mask** | **Toolbox** |
| --- | --- | --- |
| Lateral Fusiform Gyrus | Fusiform Gyrus (FG) 2 & 4 | SPM Anatomy Toolbox |
| Basolateral Amygdala | Amygdala (LB) | SPM Anatomy Toolbox |
| Anterior Cingulate Cortex | Cingulum Anterior | WFU PickAtlas |
| Dorsolateral Prefrontal Cortex | Brodmann area 46 & Frontal Inferior Triangularis | WFU PickAtlas |
| Orbitofrontal Cortex | Frontal Inferior Orbital | WFU PickAtlas |
| Insula | Insula | WFU PickAtlas |


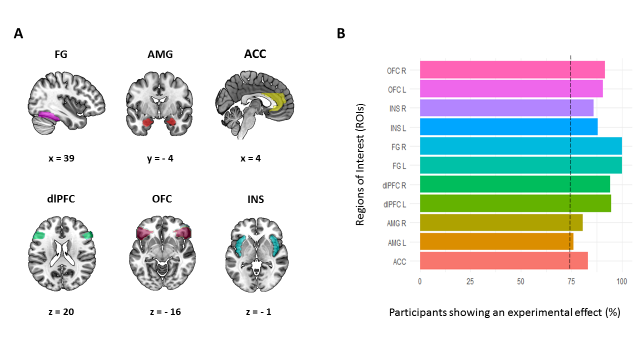


**Figure S3. Regions of interest.** The left panel shows anatomical ROI masks used for regional time series extraction. The anatomical masks were mapped on the brain surface using the MRIcroGL toolbox (<https://www.nitrc.org/projects/mricrogl>). The right panel shows the percentage of participants showing an experimental effect during the face-matching task for each ROI. The dashed line indicates that at least 75% of participants showed task-related activation/deactivation for each ROI. Abbreviations. ACC, anterior cingulate cortex; AMG, amygdala; dlPFC, dorsolateral prefrontal cortex; FG, fusiform gyrus; INS, insula; L, left; OFC, orbitofrontal cortex; R, right.

## S7. Dynamic Causal Modelling

DCM is a mathematical framework that uses the *multiple input-state-output model* to make inferences about hidden neural states underlying measured time series. The inputs correspond to the stimulus function that is designed to elicit neural activity. Inputs can elicit changes in neural activity by directly changing synaptic responses in primary sensory areas (driving input) or by altering the strength of neural coupling among brain regions in a context-dependent manner (modulatory input). The states represent neural responses and other neurophysiological variables, whereas the outputs are region-specific BOLD responses (Friston et al., 2003).

DCM embodies two distinct states: neuronal and hemodynamic states. The neuronal state constitutes neuronal dynamics of a system with k coupled brain regions. It models temporal evolution of a neuronal system over time as a function of current neural state *z*, experimental input *u,* and neural coupling parameters *θ^n^* that represent the strength of connectivity within and between brain regions.

ż = 𝑓 (z, u, θ^n^ )

Eq. (1)

The bilinear form of Eq. (1) is:

ż = (A + Σ u_j_ B^j^) z + Cu

$$A= \frac{\partial F}{\partial z}=\frac{\partial ż}{\partial z}$$

$$B= \frac{\partial^{2}F}{\partial zu_{j}}=\frac{\partial}{\partial u_{j}} \frac{\partial ż}{\partial z}$$

$$C= \frac{\partial F}{\partial u}$$

Eq. (2)

where the parameter matrix A ∈ ℝ^n × n^ represents intrinsic (i.e. context-independent) effective connectivity within and between brain regions. B^(j)^ ∈ ℝ^n × n^ is the context-sensitive changes in intrinsic connectivity modulated by experimental manipulation j and C ∈ ℝ^n × j^ is the direct influence of each of the j experimental condition on each region (Zhou et al., 2018).

After setting a biologically realistic neuronal model, the area-specific neuronal states are transformed into measured responses (i.e., predicted BOLD time series) by the hemodynamic forward model (Friston et al., 2000). The hemodynamic forward model enables to estimate the parameters of a neuronal system from the observed BOLD time series (Figure S4). The main purpose of DCM is to specify a reasonable neuronal system that minimizes the discrepancy between predicted and observed BOLD time series (Friston et al., 2003).


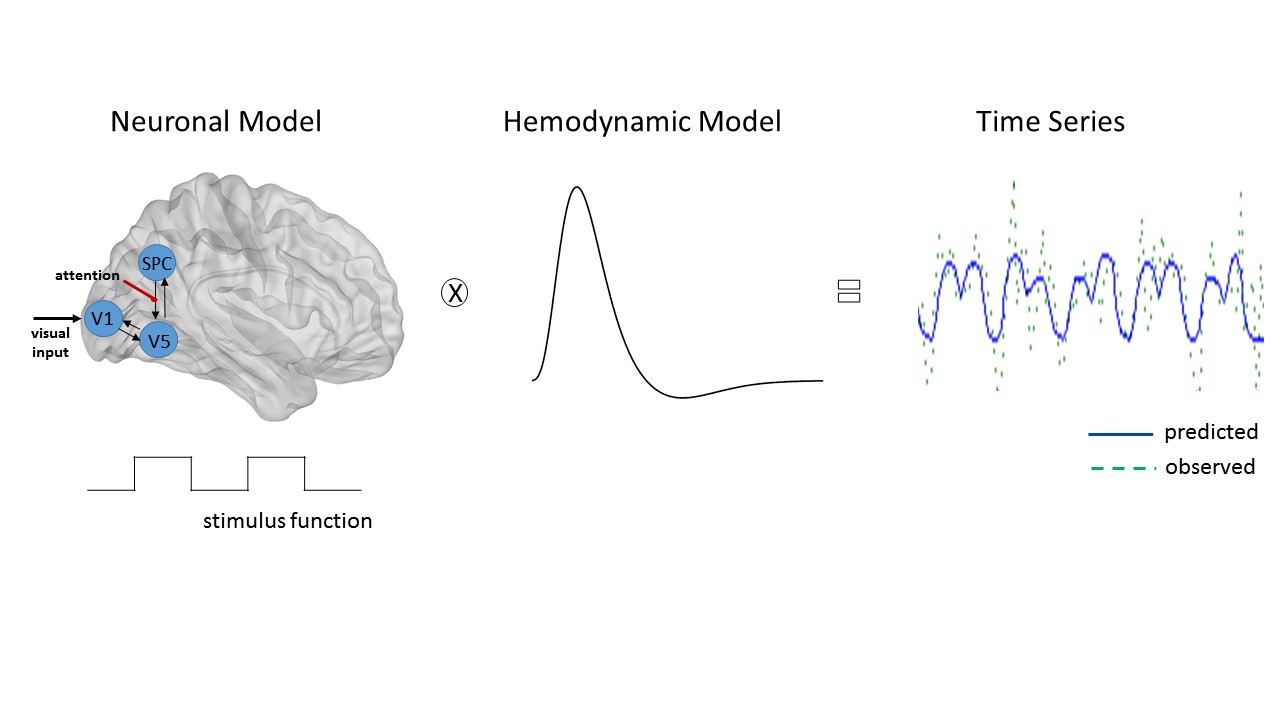


**Figure S4. DCM forward model.** DCM forward model combines a neuronal model with a hemodynamic model to generate time series from underlying causes (e.g., neural fluctuations and connection strengths). Experimental stimuli cause changes in neural activity and the resulting neural activity changes hemodynamic responses (e.g., blood flow and volume) and generation of the BOLD signal in turn. With the addition of observation noise, observed fMRI time series are obtained (Zeidman et al., 2019a). Since the generated time series by DCM is dependent on neuronal model parameters (e.g., connectivity architecture), DCM aims to find the model which explains the data best among competing models.

DCM is deemed especially useful for multifactorial designs in which one factor controls sensory perturbation, and another factor controls the context-sensitive changes (e.g., attention) (Friston et al., 2003; Stephan et al., 2010). However, DCM can reliably be applied to simple experimental designs. Several clinical studies utilized DCM with a simple experimental design that contains only one experimental condition interleaved with baseline or control task to investigate group differences in intrinsic connections (Agosta et al., 2010; Chu et al., 2018; Collignon et al., 2013; De Almeida et al., 2011; Mintzopoulos et al., 2009; Miyake et al., 2010). Similarly, since our design did not include any modulatory input, we here did not model bilinear or modulatory terms. Therefore, the contrast used for DCM analysis (faces > shapes) mainly corresponded to the effect of faces on the intrinsic (i.e., baseline) connectivity. However, since all facial stimuli carried emotional information (e.g., angry or fearful facial expression), the task implicitly allowed for the effect of emotional valence on the intrinsic connections. In this study, we estimated only two sets of parameters: 1) the direct influence of faces on regional activity and 2) the intrinsic connections within and between regions. The driving input (i.e., presentation of emotional faces) entered the model through bilateral fusiform gyri and propagated through the network via intrinsic connections (A matrix).

Since the bilinear terms were excluded, the DCM formula was written in this form:

ż = Az + Cu

Eq. (3)

where A ∈ ℝ^n × n^ represented intrinsic connections within and between regions in which input-driven local responses were propagated and C ∈ ℝ^n × J^ was the direct influence of driving (exogenous) input on each region.

To compare all possible nested models within the network, we specified a fully connected A matrix (11x11=121 neural coupling parameters, Figure S5) with self-connections on the diagonal and between regions connections on the off-diagonals. The fully-connected model allowed us to examine both intrahemispheric and interhemispheric connections that have not been previously investigated in effective connectivity studies.


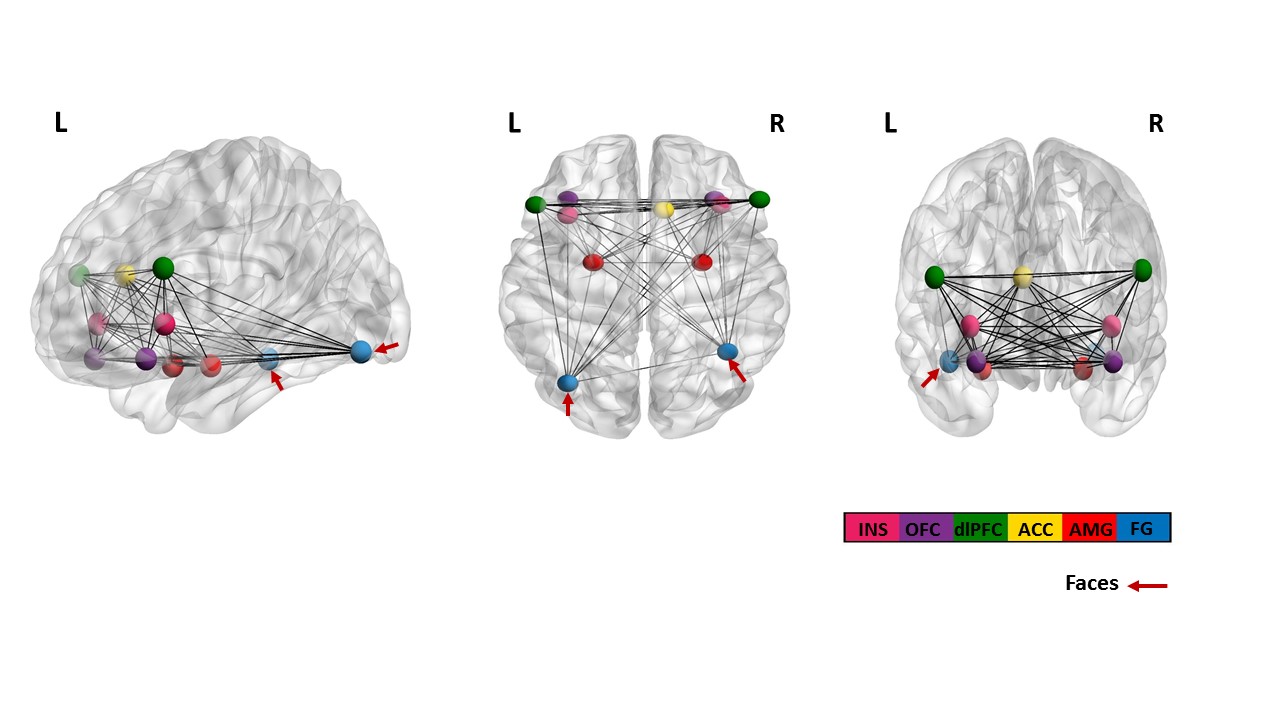


**Figure S5. Fully-connected DCM model with 121 neural coupling parameters.** The fully-connected model modelled all possible connections within (i.e., self-connections) and between regions during the face-matching task. The direct influence of faces entered the model through bilateral fusiform gyri and was propagated via intrinsic connections. Abbreviations: ACC, anterior cingulate cortex; AMG, amygdala; dlPFC, dorsolateral prefrontal cortex; FG, fusiform gyrus; INS, insula; L, left; OFC, orbitofrontal cortex; R, right.

## S8. Flow diagram

**
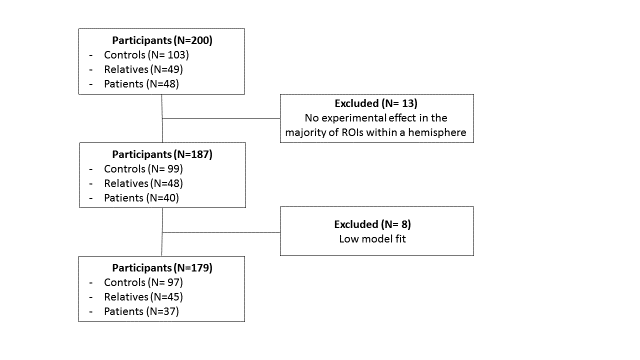
**

**Figure S6. Flow diagram of study participants.** Two-hundred participants from two multicenter studies (Erk et al., 2014; Wackerhagen et al., 2019) were selected for the current study. We excluded 13 participants who did not show an experimental effect (faces > shapes; p < .05) in more than two ROIs within a hemisphere. Furthermore, 8 participants were excluded after the estimation of subject-level DCMs due to low model fit. Second-level DCM analysis was performed with the final sample (n=179).

## S9. Empirical Bayes for Group DCM

The Parametric Empirical Bayes (PEB) is a hierarchical Bayesian framework to estimate effective connectivity parameters at the group level. It uses the full posterior density over the parameters (i.e., expected values and covariance) from subject-level DCM analysis to inform the group-level DCM results (Friston et al., 2016; Zhou et al., 2018). Since estimated parameters in DCM are probability densities, using both expected values and covariance of parameters in second-level analysis makes it possible to down-weigh participants with noisy data and uncertain parameters (Zeidman et al., 2019b).

In a PEB analysis, hypotheses about between-subject variability are encoded via a group-level design matrix, in which each row represents a subject and each column represents a regressor. As a requirement, the design matrix expects the first column to be ones to model commonalities (i.e., group mean) across participants. The following columns usually represent the effect of interests (e.g., group differences or a behavioral measure). Covariates of no interest can be placed in subsequent columns after the effect of interests. In our PEB analysis for group differences, the first column was identical and corresponded to the group mean. The second column was used to encode hypotheses about the group differences in effective connectivity (e.g., healthy controls versus patients). Age, sex, education, study site and mean head motion were included in all analyses as covariates of no interest. All covariates following the group mean were mean-centered.

Once hypotheses about between-subject variability are encoded, two different approaches are available to obtain effective connectivity parameters at the group-level. One can compare the fully-connected model with nested models (i.e., models with certain parameters switched off) to test specific hypotheses or simply prune the parameters from the full model, which did not contribute to model evidence (i.e., free energy). We here adopted the latter approach since it is challenging to test specific hypotheses with a model having a large number of connection parameters. The Bayesian model reduction was performed to prune the connection parameters that did not contribute to the model evidence. The procedure compared all nested models derived from the full model (based on its free energy) and iteratively discarded parameters that did not contribute to model evidence until discarding any parameter started to decrease model evidence (Friston et al., 2015). Finally, the parameters of the best 256 reduced models were averaged and weighted by their model evidence using Bayesian model averaging.

## S10. DCM Pipeline

Figure S7 shows the DCM pipeline that describes the main steps of DCM analyses performed in this study. At the subject level, we first specified a fully-connected model in which all possible intrinsic connections (11 x 11 = 121 neural coupling parameters) were switched on. Neural coupling parameters that explain the data best were then estimated using the Variational Laplace scheme. The estimated parameters (i.e., expected values and covariance) at the subject-level were then taken in group-level analysis to estimate neural coupling parameters at the group level. To encode between-subject variability, we specified a PEB design matrix with the following regressors: group mean, group differences (e.g., healthy controls versus patients), age, mean head motion, sex, education and study site. Once we specified the PEB design matrix, Bayesian Model Reduction was performed to compare all nested models derived from the full model and prune away connection parameters that did not contribute to model evidence (i.e., free energy). Then, the parameters of the best 256 reduced models, present in the Occam’s window, were averaged and weighted based on their model evidence using Bayesian Model Averaging. Estimated parameters at the group level (e.g., the group mean and group differences between healthy controls and patients) can be shown in the bottom right panel. Only connection parameters with posterior probability greater than 95% of being present vs. absent were shown here.


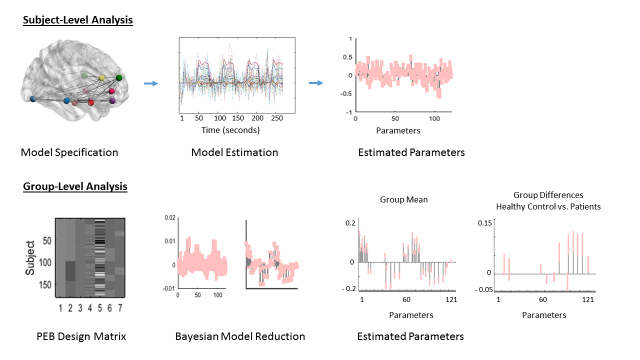


**Figure S7. DCM Pipeline for Subject- and Group-level analyses.**

# Results

## S11. Post-hoc Tests for Psychological Measurements

**Table S5. Post-hoc group comparisons for psychological measurements.**

| **Psychological Measurements** | **HCs vs. RELs** | | **HCs vs. PATs** | | **RELs vs. PATs** | |
| --- | --- | --- | --- | --- | --- | --- |
|  | F | p | F | P | F | p |
| SCL-90 Depression | -15.62 | .34 | -92.09 | <0.000 ^b^ | -76.48 | <0.000 ^b^ |
| BDI | -31.80 | .005 ^a^ | -98.98 | <0.000 ^b^ | -67.60 | <0.000 ^b^ |
| STAI-S | -.29 | .98 | -18.16 | <0.000 ^b^ | -17.87 | <0.000 ^b^ |
| STAI-T | -2.30 | .32 | -23.80 | <0.000 ^b^ | -20.77 | <0.000 ^b^ |
| NEO-FFI Neuroticism | -3.18 | .03 ^a^ | -18.21 | <0.000 ^b^ | -15.03 | <0.000 ^b^ |
| Negative Affect | -21.62 | .08 | -93.25 | <0.000 ^b^ | -71.63 | <0.000 ^b^ |

^a^ p<0.05, ^b^ p<0.001.

Abbreviations: BDI, Beck’s Depression Inventory; HCs, healthy controls; NEO-FFI, NEO-Five Factory Inventory; PATs, patients; RELs, relatives; SCL90-R Depression, Symptom Checklist 90 Revised Depression Scale; STAI-S, State Trait Anxiety Inventory - State Anxiety; STAI-T, State Trait Anxiety Inventory - Trait Anxiety

## S12. Task-Related Brain Activity

**Table S6. Anatomical locations of brain regions showing the main effect of task.**

| **Brain Region** | **k** | **T** | **MNI Coordinates** | | |
| --- | --- | --- | --- | --- | --- |
| **Faces > Shapes** |  |  |  |  |  |
| Lingual Gyrus R | 13883 | 34.97 | 18 | -88 | -7 |
| Cuneus R |  | 33.95 | 21 | -94 | 11 |
| Calcarine Sulcus L |  | 33.35 | -12 | -91 | -7 |
| Inferior Occipital Gyrus L |  | 33.18 | -24 | -94 | -4 |
| Calcarine Sulcus R |  | 31.69 | 15 | -97 | 2 |
| Fusiform L |  | 29.69 | -36 | -73 | -13 |
| Fusiform R |  | 28.29 | 39 | -55 | -16 |
| Inferior Occipital Gyrus R |  | 28.18 | 33 | -73 | -10 |
| Thalamus L |  | 26.66 | -24 | -31 | 2 |
| Thalamus R |  | 24.88 | 24 | -31 | 2 |
| Amygdala R |  | 20.19 | 21 | -4 | -13 |
| Amygdala L |  | 19.02 | -21 | -7 | -13 |
| Inferior Frontal Gyrus (Pars Triangularis) R |  | 17.59 | 54 | 32 | 20 |
| Hippocampus L |  | 17.58 | -21 | -10 | -13 |
| Hippocampus R |  | 15.91 | 30 | -10 | -13 |
| Inferior Frontal Gyrus (Orbital) R |  | 14.73 | 33 | 32 | -16 |
| Inferior Frontal Gyrus (Pars Triangularis) L |  | 13.39 | -51 | 29 | 23 |
| Middle Frontal Gyrus L |  | 12.70 | -48 | 26 | 35 |
| Inferior Frontal Gyrus (Orbital) L |  | 12.19 | -36 | 32 | -16 |
| Precentral Gyrus L |  | 11.52 | -36 | 8 | 29 |
| Superior Temporal Gyrus R |  | 10.30 | 45 | -40 | 11 |
| Superior Frontal Gyrus L |  | 10.20 | -12 | 56 | 35 |
| Cerebellum R |  | 10.03 | 12 | -76 | -34 |
| Cerebellum L |  | 9.86 | -18 | -43 | -43 |
| Insula R |  | 9.00 | 36 | 29 | -1 |
| Insula L |  | 8.10 | -36 | 23 | -1 |
| Supplementary Motor Area R | 91 | 6.30 | 6 | 20 | 65 |
| Supplementary Motor Area L |  | 5.84 | -6 | 17 | 50 |
| **Shapes > Faces** |  |  |  |  |  |
| Inferior Parietal Lobe L | 315 | 11.12 | -51 | -31 | 38 |
| Inferior Parietal Lobe R | 646 | 10.84 | 57 | -55 | 41 |
| Angular Gyrus R | 897 | 10.32 | 60 | -52 | 35 |
| Supramarginal Gyrus R |  | 10.04 | 60 | -46 | 11 |
| Postcentral Gyrus R |  | 9.48 | 48 | -28 | 41 |
| Supramarginal Gyrus L |  | 9.21 | -57 | -31 | 38 |
| Middle Cingulate Cortex |  | 9.16 | 6 | -25 | 38 |
| Postcentral Gyrus L |  | 8.40 | -42 | -34 | 41 |
| Precuneus R |  | 8.03 | 24 | -46 | 8 |
| Angular Gyrus L |  | 7.18 | -48 | -73 | 32 |
| Middle Occipital Cortex | 34 | 8.66 | -42 | -76 | 38 |
| Middle Frontal Gyrus L | 176 | 7.19 | -24 | 32 | 29 |
| Anterior Cingulate Cortex | 246 | 7.00 | 9 | 26 | 20 |
| Middle Frontal Gyrus R | 160 | 6.44 | 27 | 29 | 35 |

p < .05 (whole brain FWE-corrected)

**Table S7. Anatomical locations of activation differences between patients, relatives and healthy controls**

| **Brain Region** | **k** | **T** | **p** | **MNI Coordinates** | | | | |
| --- | --- | --- | --- | --- | --- | --- | --- | --- |
| **Controls > Patients** |  |  |  |  | |  |  | |
| Superior Occipital Cortex R (BA 18) | 450 | 6.26 | < 0.05 ^a^ | | 15 | -88 | | 17 |
| Superior Occipital Cortex L |  | 5.94 | < 0.05 ^a^ | | -15 | -85 | | 17 |
| Lingual Gyrus L |  | 5.81 | < 0.05 ^a^ | | -9 | -79 | | -1 |
| Lingual Gyrus R |  | 5.52 | < 0.05 ^a^ | | 16 | -79 | | 2 |
| Fusiform L | 32 | 4.81 | < 0.001 ^b^ | | -30 | -64 | | -4 |
| Superior Parietal Lobe L | 52 | 4.32 | < 0.001 ^b^ | | -27 | -64 | | 59 |
| Fusiform R | 24 | 4.00 | < 0.001 ^b^ | | 24 | -70 | | -7 |
| Thalamus L | 28 | 3.83 | < 0.001 ^b^ | | -15 | -34 | | 5 |
| Middle Frontal Gyrus L (BA 6) | 11 | 3.67 | < 0.001 ^b^ | | -39 | 11 | | 53 |
| Superior Temporal Gyrus L | 37 | 3.56 | < 0.001 ^b^ | | -45 | -49 | | 14 |
| Inferior Frontal Gyrus R (BA 45) | 16 | 3.41 | < 0.001 ^b^ | | 51 | 26 | | 23 |
| **Relatives > Patients** |  |  |  | |  |  | |  |
| Calcarine Sulcus L | 621 | 8.09 | < 0.05 ^a^ | | 0 | -85 | | 8 |
| Lingual Gyrus L |  | 7.10 | < 0.05 ^a^ | | -3 | -82 | | 2 |
| Lingual Gyrus R |  | 6.88 | < 0.05 ^a^ | | 12 | -76 | | -4 |
| Superior Occipital Gyrus L |  | 5.70 | < 0.05 ^a^ | | -24 | -82 | | 26 |
| Inferior Occipital Gyrus L |  | 5.45 | < 0.05 ^a^ | | -39 | -82 | | -7 |
| Fusiform L | 22 | 5.62 | < 0.001 ^b^ | | -24 | -70 | | -7 |
| Fusiform R | 27 | 4.37 | < 0.001 ^b^ | | 24 | -70 | | -7 |
| Superior Temporal Gyrus L | 43 | 4.12 | < 0.001 ^b^ | | -45 | -49 | | 14 |
| Inferior Frontal Gyrus L | 33 | 4.08 | < 0.001 ^b^ | | -33 | 14 | | 23 |
| Superior Parietal Lobe L | 69 | 3.95 | < 0.001 ^b^ | | -24 | -64 | | 56 |
| Inferior Frontal Gyrus R (BA 45) | 17 | 3.89 | < 0.001 ^b^ | | 55 | 51 | | 17 |
| Middle Frontal Gyrus L (BA 6) | 30 | 3.66 | < 0.001 ^b^ | | -33 | 11 | | 53 |
| Precentral Gyrus R (BA6) | 41 | 3.65 | < 0.001 ^b^ | | 12 | 36 | | -4 |
| Inferior Frontal Gyrus (Orbital) R | 24 | 3.38 | < 0.001 ^b^ | | 36 | 29 | | -10 |
| **Relatives > Controls** |  |  |  | |  |  | |  |
| Lingual Gyrus L | 46 | 4.46 | < 0.001 ^b^ | | -18 | -67 | | -7 |
| Calcarine Sulcus L | 38 | 4.30 | < 0.001 ^b^ | | 0 | -88 | | 8 |
| Lingual Gyrus R | 23 | 3.84 | < 0.001 ^b^ | | 12 | -73 | | -10 |
| Middle Frontal Gyrus R | 10 | 3.39 | < 0.001 ^b^ | | 30 | 35 | | 38 |

^a^ FWE corrected, ^b^ uncorrected

## S13. Group Differences in Effective Connectivity

**Table S8. Between-group differences of effective connectivity during the face-matching task.**

|  | **Connection Parameter** | **Effect size**  **(Posterior Expectation)** | **95% Bayesian Confidence Interval** |
| --- | --- | --- | --- |
| **Controls > Patients** |  |  |  |
|  | AMG R 🡪 FG R | 0.04 | [0.01, 0.06] |
|  | AMG R 🡪 FG L | 0.03 | [-0.001, 0.05] |
|  | OFC R 🡪 INS L | 0.03 | [0.01, 0.05] |
|  | AMG L 🡪 FG R | 0.07 | [0.04, 0.10] |
|  | OFC L 🡪 FG R | 0.07 | [0.05, 0.10] |
|  | OFC L 🡪 FG L | 0.08 | [0.05, 0.12] |
|  | dlPFC L 🡪 FG R | 0.09 | [0.06, 0.12] |
|  | dlPFC L🡪 OFC R | 0.04 | [0.01, 0.06] |
|  | dlPFC L 🡪 dlPFC R | 0.03 | [0.00, 0.06] |
|  | dlPFC L 🡪 FG L | 0.09 | [0.06, 0.12] |
|  | dlPFC L 🡪 AMG L | 0.03 | [-0.001, 0.06] |
|  | dlPFC L 🡪 INS L | 0.02 | [-0.01, 0.05] |
| **Controls < Patients** |  |  |  |
|  | dlPFC R 🡪 FG R | -0.02 | [-0.05, 0.001] |
|  | AMG L 🡪 INS L | -0.02 | [-0.05, -0.000] |
| **Relatives > Patients** |  |  |  |
|  | ACC 🡪 dlPFC L | 0.03 | [0.01, 0.05] |
|  | ACC 🡪 INS L | 0.02 | [0.000, 0.03] |
|  | AMG L 🡪 FG R | 0.06 | [0.03, 0.09] |
|  | OFC L 🡪 FG L | 0.05 | [0.02, 0.08] |
|  | dlPFC L 🡪 FG R | 0.08 | [0.05, 0.12] |
|  | INS L 🡪 FG R | 0.07 | [0.04, 0.10] |
|  | INS L 🡪 OFC L | 0.05 | [0.02, 0.08] |
| **Controls > Relatives** |  |  |  |
|  | OFC R 🡪 FG L | 0.04 | [0.02, 0.06] |
|  | OFC R 🡪 INS L | 0.02 | [0.00, 0.04] |
|  | OFC L 🡪 FG R | 0.04 | [0.01, 0.06] |
| **Controls < Relatives** |  |  |  |
|  | ACC 🡪 AMG L | -0.01 | [-0.02, 0.001] |
|  | ACC 🡪 dlPFC L | -0.02 | [-0.04, -0.003] |
|  | FG L 🡪 FG R | -0.03 | [-0.05, -0.01] |

Abbreviations. ACC, anterior cingulate cortex; AMG, amygdala; dlPFC, dorsolateral prefrontal cortex; FG, fusiform gyrus; INS, insula; L, left; OFC, orbitofrontal cortex; R, right.

## S14. Medication Effect

To examine the potential effect of antidepressant treatment on task-related effective connectivity, we included the medication status as a covariate of no-interest in the PEB design matrix in addition to other covariates. The value 1 is assigned to patients who are under antidepressant treatment, whereas the value 0 is assigned to all participants who do not take any psychotropic medication. The values are then mean-centered. Results are shown in Figure S8. All reported results were based on the criterion posterior probability (free energy with versus without parameter) are larger than 0.95.


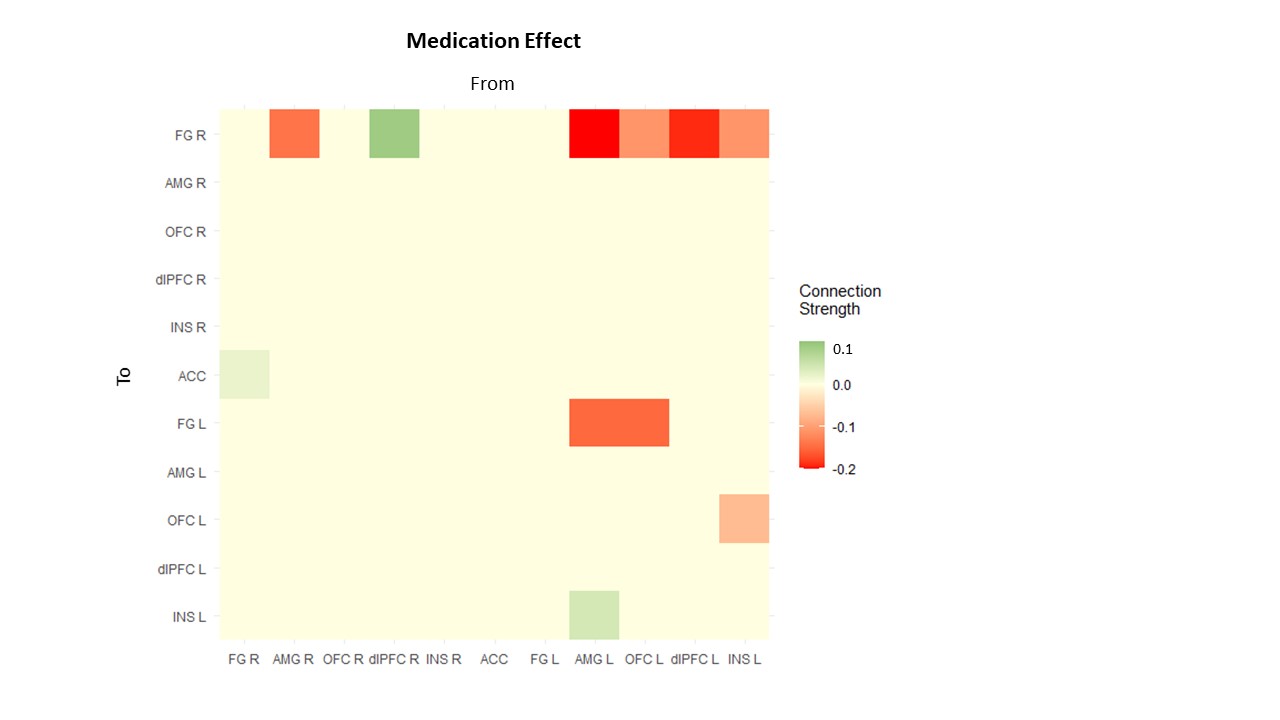


**Figure S8. The effect of medication on effective connections.** Green colors indicate positive associations between effective connection strength and medication status, whereas red colors indicate negative associations between effective connection strengths and medication status (posterior probability > 0.95). Abbreviations. ACC, anterior cingulate cortex; AMG, amygdala; dlPFC, dorsolateral prefrontal cortex; FG, fusiform gyrus; INS, insula; L, left; OFC, orbitofrontal cortex; R, right.

## S15. Brain-Behavior Relationship

**Table S9. The associations between negative affect scores and effective connectivity during the face-matching task (posterior probability [free energy with versus without] > .95).**

|  | **Connection Parameter** | **Effect Size (Posterior Expectation)** | **95% Bayesian Confidence Interval** |
| --- | --- | --- | --- |
| **Controls** | | | |
|  | AMG R 🡪 FG R | 0.06 | [0.058- 0.061] |
|  | OFC R 🡪 AMG R | 0.08 | [0.068- 0.092] |
|  | OFC R 🡪 AMG L | 0.11 | [0.108- 0.111] |
|  | INS R 🡪 INS R | 0.11 | [0.109- 0.111] |
|  | INS R 🡪 ACC | -0.06 | [-0.06- -0.06] |
|  | INS R 🡪 dlPFC L | 0.15 | [0.149- 0.151] |
|  | ACC 🡪 INS R | -0.03 | [-0.038- -0.027] |
|  | FG L 🡪 dlPFC L | -0.12 | [-0.121- -0.119] |
|  | OFC L 🡪 dlPFC L | 0.11 | [0.107- 0.113] |
| **Relatives** | | | |
|  | FG R 🡪 FG R | 0.32 | [0.284- 0.356] |
|  | FG R 🡪 AMG R | 0.07 | [0.069- 0.071] |
|  | FG R 🡪 FG L | 0.19 | [0.187- 0.191] |
|  | AMG R 🡪 FG L | 0.03 | [0.026- 0.034] |
|  | AMG R 🡪 OFC L | 0.05 | [0.049- 0.051] |
|  | OFC R 🡪 ACC | -0.03 | [-0.031- -0.029] |
|  | dlPFC R 🡪 FG R | -0.14 | [-0.143- -0.137] |
|  | dlPFC R 🡪 AMG R | -0.07 | [-0.071- -0.069] |
|  | dlPFC R 🡪 FG L | -0.12 | [-0.122- -0.118] |
|  | dlPFC R 🡪 INS L | -0.10 | [-0.102- -0.098] |
|  | ACC 🡪 FG R | -0.42 | [-0.424- -0.416] |
|  | ACC 🡪 ACC | 0.17 | [0.165- 0.175] |
|  | ACC 🡪 FG L | -0.16 | [-0.161- -0.158] |
|  | FG L 🡪 ACC | 0.11 | [0.109- 0.11] |
|  | dlPFC L 🡪 ACC | -0.19 | [-0.194- -0.186] |
|  | dlPFC L 🡪 INS L | -0.12 | [-0.122- -0.118] |
|  | INS L 🡪 FG L | -0.03 | [-0.031- -0.029] |
|  | INS L 🡪 dlPFC L | -0.10 | [-0.102- -0.098] |
|  | | | |
| **Table S9** (continued) |  |  |  |
| **Patients** |  |  |  |
|  | FG R 🡪 dlPFC L | 0.07 | [0.069- 0.071] |
|  | OFC R 🡪 INS R | 0.04 | [0.039- 0.041] |
|  | dlPFC R 🡪 dlPFC L | 0.07 | [0.069- 0.071] |
|  | INS R 🡪 FG R | -0.04 | [-0.041- -0.039] |
|  | ACC 🡪 FG L | -0.09 | [-0.091- -0.089] |
|  | ACC 🡪 dlPFC L | 0.13 | [0.128- 0.132] |
|  | FG L 🡪 AMG R | -0.02 | [-0.21- -0.19] |
|  | FG L 🡪 ACC | -0.07 | [-0.071- -0.069] |
|  | AMG L 🡪 FG R | -0.08 | [-0.081- -0.079] |
|  | AMG L 🡪 AMG R | 0.02 | [0.019- 0.21] |
|  | AMG L 🡪 dlPFC L | -0.04 | [-0.041- -0.039] |
|  | AMG L 🡪 INS L | 0.07 | [0.069- 0.071] |
|  | OFC L 🡪 FG R | -0.11 | [-0.111- -0.109] |
|  | OFC L 🡪 ACC | 0.06 | [0.059- 0.061] |
|  | OFC L 🡪 INS L | 0.06 | [0.059- 0.061] |
|  | dlPFC L 🡪 FG R | 0.08 | [0.079- 0.081] |
|  | dlPFC L 🡪 INS L | 0.03 | [0.029- 0.031] |
|  | INS L 🡪 dlPFC R | -0.02 | [-0.021- -0.019] |

Abbreviations. ACC, anterior cingulate cortex; AMG, amygdala; dlPFC, dorsolateral prefrontal cortex; FG, fusiform gyrus; INS, insula; L, left; OFC, orbitofrontal cortex; R, right.

# References

Agosta, F., Rocca, M. A., Pagani, E., Absinta, M., Magnani, G., Marcone, A., Falautano, M., Comi, G., Gorno-Tempini, M. L., & Filippi, M. (2010). Sensorimotor network rewiring in mild cognitive impairment and Alzheimer’s disease. *Human Brain Mapping*. https://doi.org/10.1002/hbm.20883

Chu, R., Meltzer, J. A., & Bitan, T. (2018). Interhemispheric interactions during sentence comprehension in patients with aphasia. *Cortex*. https://doi.org/10.1016/j.cortex.2018.08.022

Collignon, O., Dormal, G., Albouy, G., Vandewalle, G., Voss, P., Phillips, C., & Lepore, F. (2013). Impact of blindness onset on the functional organization and the connectivity of the occipital cortex. *Brain*. https://doi.org/10.1093/brain/awt176

De Almeida, J. R. C., Kronhaus, D. M., Sibille, E. L., Langenecker, S. A., Versace, A., LaBarbara, E. J., & Phillips, M. L. (2011). Abnormal left-sided orbitomedial prefrontal cortical-amygdala connectivity during happy and fear face processing: A potential neural mechanism of female MDD. *Frontiers in Psychiatry*. https://doi.org/10.3389/fpsyt.2011.00069

Eickhoff, S. B., Stephan, K. E., Mohlberg, H., Grefkes, C., Fink, G. R., Amunts, K., & Zilles, K. (2005). A new SPM toolbox for combining probabilistic cytoarchitectonic maps and functional imaging data. *NeuroImage*. https://doi.org/10.1016/j.neuroimage.2004.12.034

Erk, S., Meyer-Lindenberg, A., Schmierer, P., Mohnke, S., Grimm, O., Garbusow, M., Haddad, L., Poehland, L., Mühleisen, T. W., Witt, S. H., Tost, H., Kirsch, P., Romanczuk-Seiferth, N., Schott, B. H., Cichon, S., Nöthen, M. M., Rietschel, M., Heinz, A., & Walter, H. (2014). Hippocampal and frontolimbic function as intermediate phenotype for psychosis: Evidence from healthy relatives and a common risk variant in cacna1c. *Biological Psychiatry*. https://doi.org/10.1016/j.biopsych.2013.11.025

Friston, K. J., Harrison, L., & Penny, W. (2003). Dynamic causal modelling. *NeuroImage*. https://doi.org/10.1016/S1053-8119(03)00202-7

Friston, K. J., Mechelli, A., Turner, R., & Price, C. J. (2000). Nonlinear responses in fMRI: The balloon model, Volterra kernels, and other hemodynamics. *NeuroImage*. https://doi.org/10.1006/nimg.2000.0630

Friston, K., Zeidman, P., & Litvak, V. (2015). Empirical Bayes for DCM: A Group Inversion Scheme. *Frontiers in Systems Neuroscience*. https://doi.org/10.3389/fnsys.2015.00164

Friston, Karl J., Litvak, V., Oswal, A., Razi, A., Stephan, K. E., Van Wijk, B. C. M., Ziegler, G., & Zeidman, P. (2016). Bayesian model reduction and empirical Bayes for group (DCM) studies. *NeuroImage*. https://doi.org/10.1016/j.neuroimage.2015.11.015

Karamizadeh, S., Abdullah, S. M., Manaf, A. A., Zamani, M., & Hooman, A. (2013). An Overview of Principal Component Analysis. *Journal of Signal and Information Processing*. https://doi.org/10.4236/jsip.2013.43b031

Mintzopoulos, D., Astrakas, L. G., Khanicheh, A., Konstas, A. A., Singhal, A., Moskowitz, M. A., Rosen, B. R., & Tzika, A. A. (2009). Connectivity alterations assessed by combining fMRI and MR-compatible hand robots in chronic stroke. *NeuroImage*. https://doi.org/10.1016/j.neuroimage.2009.03.007

Miyake, Y., Okamoto, Y., Onoda, K., Shirao, N., Okamoto, Y., Otagaki, Y., & Yamawaki, S. (2010). Neural processing of negative word stimuli concerning body image in patients with eating disorders: An fMRI study. *NeuroImage*. https://doi.org/10.1016/j.neuroimage.2009.12.095

Power, J. D., Barnes, K. A., Snyder, A. Z., Schlaggar, B. L., & Petersen, S. E. (2012). Spurious but systematic correlations in functional connectivity MRI networks arise from subject motion. *NeuroImage*. https://doi.org/10.1016/j.neuroimage.2011.10.018

Power, J. D., Mitra, A., Laumann, T. O., Snyder, A. Z., Schlaggar, B. L., & Petersen, S. E. (2014). Methods to detect, characterize, and remove motion artifact in resting state fMRI. *NeuroImage*. https://doi.org/10.1016/j.neuroimage.2013.08.048

Stephan, K. E., Penny, W. D., Moran, R. J., den Ouden, H. E. M., Daunizeau, J., & Friston, K. J. (2010). Ten simple rules for dynamic causal modeling. In *NeuroImage*. https://doi.org/10.1016/j.neuroimage.2009.11.015

Wackerhagen, C., Veer, I. M., Erk, S., Mohnke, S., Lett, T. A., Wüstenberg, T., Romanczuk-Seiferth, N. Y., Schwarz, K., Schweiger, J. I., Tost, H., Meyer-Lindenberg, A., Heinz, A., & Walter, H. (2019). Amygdala functional connectivity in major depression-disentangling markers of pathology, risk and resilience. *Psychological Medicine*. https://doi.org/10.1017/S0033291719002885

Wackerhagen, C., Wüstenberg, T., Mohnke, S., Erk, S., Veer, I. M., Kruschwitz, J. D., Garbusow, M., Romund, L., Otto, K., Schweiger, J. I., Tost, H., Heinz, A., Meyer-Lindenberg, A., Walter, H., & Romanczuk-Seiferth, N. (2017). Influence of Familial Risk for Depression on Cortico-Limbic Connectivity during Implicit Emotional Processing. *Neuropsychopharmacology*. https://doi.org/10.1038/npp.2017.59

Zeidman, P., Jafarian, A., Seghier, M. L., Litvak, V., Cagnan, H., Cathy, J., Friston, K. J., & Wing, W. (2019a). A tutorial on group effective connectivity analysis, part 1: first level analysis with DCM for fMRI. *Neuroimage*. <https://doi.org/10.1016/j.neuroimage.2019.06.031>

Zeidman, P., Jafarian, A., Seghier, M. L., Litvak, V., Cagnan, H., Price, C. J., & Friston, K. J. (2019b). A guide to group effective connectivity analysis, part 2: Second level analysis with PEB. *NeuroImage*. https://doi.org/10.1016/j.neuroimage.2019.06.032

Zhou, Y., Zeidman, P., Wu, S., Razi, A., Chen, C., Yang, L., Zou, J., Wang, G., Wang, H., & Friston, K. J. (2018). Altered intrinsic and extrinsic connectivity in schizophrenia. *NeuroImage: Clinical*. https://doi.org/10.1016/j.nicl.2017.12.006
